# Benthic Microbial Communities in a Seasonally Ice-Covered Sub-Arctic River (Pasvik River, Norway) Are Shaped by Site-Specific Environmental Conditions

**DOI:** 10.3390/microorganisms10051022

**Published:** 2022-05-12

**Authors:** Maria Papale, Carmen Rizzo, Stefania Giannarelli, Gabriella Caruso, Stefano Amalfitano, Paul Eric Aspholm, Giovanna Maimone, Stefano Miserocchi, Alessandro Ciro Rappazzo, Angelina Lo Giudice, Maurizio Azzaro

**Affiliations:** 1 Institute of Polar Sciences, National Research Council (CNR-ISP), Spianata S. Raineri 86, 98122 Messina, Italy; maria.papale@isp.cnr.it (M.P.); carmen.rizzo@szn.it (C.R.); gabriella.caruso@cnr.it (G.C.); giovanna.maimone@cnr.it (G.M.); alessandro.rappazzo@unive.it (A.C.R.); maurizio.azzaro@cnr.it (M.A.); 2 Department of Marine Biotechnology, Zoological Station “Anton Dohrn”, Villa Pace, Contrada Porticatello, 98168 Messina, Italy; 3 Department of Chemistry and Industrial Chemistry, University of Pisa, Via Giuseppe Moruzzi 13, 56124 Pisa, Italy; stefania.giannarelli@unipi.it; 4 Water Research Institute, National Research Council (CNR-IRSA), Via Salaria Km 29,300, Monterotondo, 00015 Rome, Italy; stefano.amalfitano@cnr.it; 5 Norwegian Institute of Bioeconomy Research (NIBIO), Svanhovd, 9925 Svanvik, Norway; paul.eric.aspholm@nibio.no; 6 Institute of Polar Sciences, National Research Council (CNR-ISP), Via Gobetti 101, 40129 Bologna, Italy; stefano.miserocchi@cnr.it; 7 Department of Environmental Sciences, Informatics and Statistics, University of Venice Ca’ Foscari, Dorsoduro 3246, 30123 Venice, Italy

**Keywords:** anthropogenic contamination, microbial community, bacterial diversity, Arctic river

## Abstract

The Pasvik River experiences chemical, physical, and biological stressors due to the direct discharges of domestic sewage from settlements located within the catchment and runoff from smelter and mine wastes. Sediments, as a natural repository of organic matter and associated contaminants, are of global concern for the possible release of pollutants in the water column, with detrimental effects on aquatic organisms. The present study was aimed at characterizing the riverine benthic microbial community and evaluating its ecological role in relation to the contamination level. Sediments were sampled along the river during two contrasting environmental periods (i.e., beginning and ongoing phases of ice melting). Microbial enzymatic activities, cell abundance, and morphological traits were evaluated, along with the phylogenetic community composition. Amplified 16S rRNA genes from bacteria were sequenced using a next-generation approach. Sediments were also analyzed for a variety of chemical features, namely particulate material characteristics and concentration of polychlorobiphenyls, polycyclic aromatic hydrocarbons, and pesticides. Riverine and brackish sites did not affect the microbial community in terms of main phylogenetic diversity (at phylum level), morphometry, enzymatic activities, and abundance. Instead, bacterial diversity in the river sediments appeared to be influenced by the micro-niche conditions, with differences in the relative abundance of selected taxa. In particular, our results highlighted the occurrence of bacterial taxa directly involved in the C, Fe, and N cycles, as well as in the degradation of organic pollutants and toxic compounds.

## 1. Introduction

Freshwater ecosystems in the Arctic region are experiencing rapid environmental modifications. Global warming is recognized as the major factor affecting ecosystem status, along with anthropogenic point and non-point pollution events. As key players in all biogeochemical cycles, mainly through organic matter degradation and nutrient remineralization, aquatic microorganisms are directly involved in the maintenance of environmental quality. They can rapidly respond to contaminated environments by developing different adaptation strategies. The transfer of harmful compounds to higher trophic levels is first microbially driven. Thus, any change in microbial communities derived from altering either the nutrient sources and availability or the physical-chemical environment will have direct repercussions on ecosystem health [1]. Anthropogenic pressure (e.g., direct discharges of domestic and agricultural wastewater, runoff from industries and mines) on freshwater systems is increasing in polar areas, thus implying microbial biomass loss, biodiversity, and function alterations with the consequent environmental deterioration. Examining the co-occurrence of contaminants and the resident microbial community will result in the identification of ecological processes and microbial interaction patterns (e.g., ecological traits, sharing of niche spaces, and ecophysiology) in response to anthropogenic perturbations [2,3].

The Pasvik River, in the Northern Fennoscandia, is subjected to multiple stressors that encompass chemical, physical, and biological factors due to the direct discharge of domestic sewages from Russian, Norwegian, and Finnish settlements located in the river catchment, runoff from smelter and mine wastes, and emissions by the foundry Pechenganikel (e.g., dust, heavy metals, and sulfur dioxide), located in the Russian town of Nikel (founded in the 1930s by the Finnish company Petsamon Nikkeli and finally closed at the end of 2020). The environmental impacts of the Pechenganikel mining and metallurgical industries are evident in terrestrial and aquatic ecosystems in the region [4]. Previous studies have mostly highlighted the influence of local heavy metal inputs (mainly Ni, Cu, Cd, Cr, Zn, As, Hg) into the sediment and biota of the Pasvik area [4,5,6,7,8,9,10]. Persistent organic pollutants (POPs), with well-known cancerogenic and mutagenic effects on organisms, have received less attention [11].

In this study, we provide integrated insight into anthropogenic and natural factors shaping the benthic microbial community of the Pasvik River. Inter- and intra-site variability of microbial community characteristics were also examined during two contrasting environmental periods (i.e., beginning and ongoing phases of ice melting). We hypothesized that (i) the functional composition and structure of microbial communities will shift under anthropogenic contamination and ice-melting conditions, and (ii) multiple contaminants will affect microbial network interactions among different phylogenetic groups.

## 2. Materials and Methods

### 2.1. Sampling Area

The Pasvik River is the largest river system in Northern Fennoscandia, flowing from Lake Inari (Finland) into the Bøkfjorden of the Barents Sea. It marks the boundary between Russia and Norway throughout its almost whole length, except for its northernmost segment. The Norwegian–Russian sector of the river system is 120 km long, has a total water-covered area of 142 km^2^, a catchment basin of 18.404 km^2^, and a mean waterflow of about 175 m^3^/s [12]. The river is shallow, ranging from 1 to 8 m in depth, with small water level fluctuations (<80 cm). Tides affect the river up to 4 km upstream of its mouth [9]. The ice-free season lasted from May/June to October/November. Geologically, the area is characterized by bedrock, with the watercourse surrounded by birch- and pinewood landscape with sectors of bobs and mires.

### 2.2. Sample Collection

**Figure 1:**
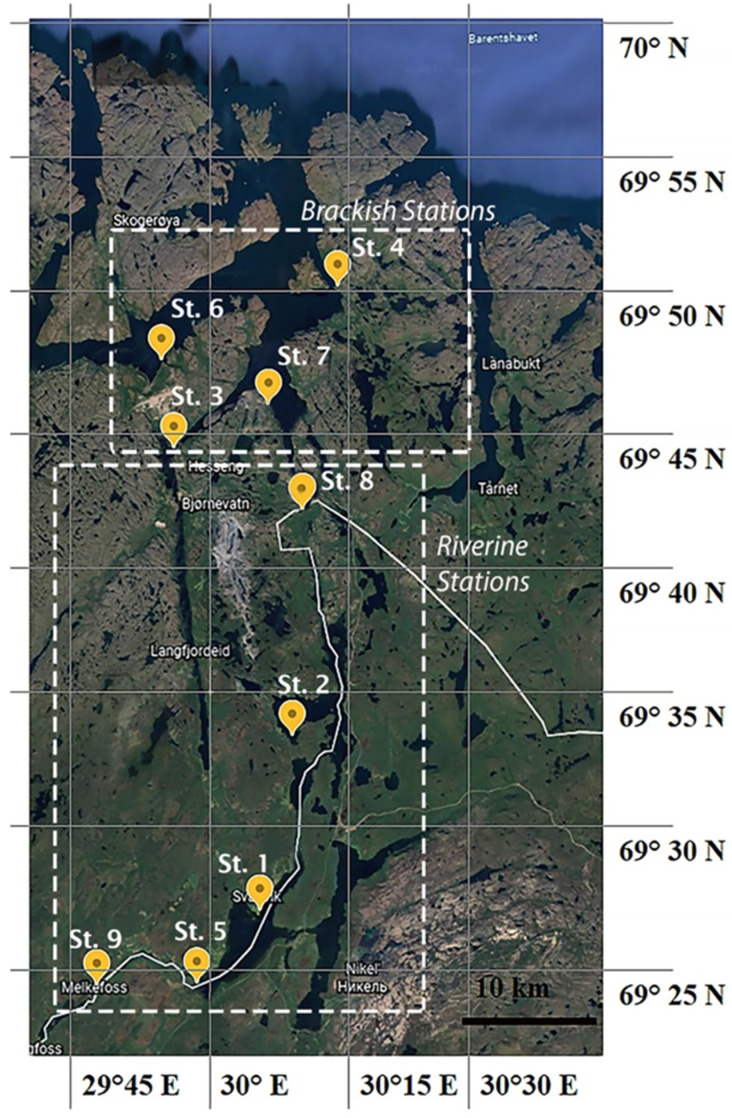
Sampling area and stations (Pasvik River).

Sediment samples (25–70 cm depth; except for St. 6) were manually collected from nine stations along the Pasvik River at the beginning and ongoing ice-melting [(i.e., Ice-melt(−): 22–29 May; (Ice-melt(+): 17–24 July)] [10,13] (Figure 1). The stations were classified as riverine (Stations 9, 5, 1, 2, and 8) or brackish (Stations 3, 7, 6, and 4). Samples for chemical analyses were collected using a pre-cleaned stainless-steel bailer and stored at −20 °C in pre-cleaned glass jars until analysis. Samples for microbiological analyses were collected using pre-sterilized polycarbonate containers and processed approximately 2 h after sampling in the laboratory of the NIBIO Svanhovd Research Station (Svanvik, Pasvik Valley). Sediments were mainly composed of sand (granulometry fraction between approximately 0.062 and 2 mm) and mud (fraction < 62 µm) [10]. The major physical–chemical water parameters have been reported elsewhere [10,13].

### 2.3. Particulate Material in Sediment Samples

H_2_O_2_ was used to remove organic matter for grain size determination. Wet sieving at 63 μm was applied to separate the sand (SAND) from silt and clay (MUD) fractions. Aliquot of sediment was freeze dried and homogenized. Elemental analysis (EA), using a CHNS Elemental Analyzer (Thermo Fisher Flash 2000 IRMS), of combusted aliquots was adopted to determine total nitrogen (N-TOT) and organic carbon (C-ORG) contents. The inorganic carbon from subsamples to be used for C-ORG quantification was removed by a 1.5 M HCl treatment [14]. Inorganic carbon was determined by subtracting the C-ORG content from the measured total carbon content (C-TOT), assuming that it is composed mainly of calcium carbonate [15]. Stable isotopic analyses of C-ORG (δ^13^C) were carried out using a FINNIGAN Delta Plus mass spectrometer directly coupled to the EA. Stable isotope data are expressed in ‰ relative to the variation (δ) from the international PDB standard [16].

### 2.4. Determination of Persistent Organic Pollutants (POPs)

The analytical procedure used for the determination of POPs involves the extraction of sediments with a suitable solvent, the reduction to an appropriate final volume, and finally, the instrumental gas-chromatographic analysis, essential when determining different classes of analytes at extremely low concentration levels [17,18]. N-hexane, isooctane, acetone, and 2-propanol (pesticides grade) were from the companies Romil, Baker Analyzed, Carlo Erba, and Sigma Aldrich, respectively. Tetrabutylammonium bisulfite (TBA) and sodium bisulfite were Sigma-Aldrich products.

Certified standard PAH solutions were from Wellington Laboratories (Guelph, ON, Canada). The National Institute of Standard and Technology (Gaithersburg, MD, USA) provided a PCB solution used for the preparation of the calibration solutions (NIST 2262), and O2Si Smart Solutions, North Charleston, SC, USA) provided certified standard solutions for OCPs.

Two different solutions, containing different mixtures of 13C and D-labeled analytes, were added before carrying out the extraction (to evaluate the extraction step recovery) and just before the analysis (to evaluate the reproducibility of the instrumental analysis stage).

Standard calibration solutions for PAHs, PCBs, and OCPs were prepared by adding the certified solutions O2Si, NIST 2262, and PAHmix to isooctane.

Ultrasonic bath (Sonorex super 10p) was supplied by Bandelin (Berlin, Germany), centrifuge (Rotofix 32 A) by Hettich (Milan, Italy), centrifugal evaporator (RC10.22 Sensitive Bio) was from the Jouan Italia company (Cologno Monzese, Milan, Italy), magnetic stirrer from VELP Scientifica (Usmate Velate, Italy), analytical scales (Als 220-4) was a Kern product (Balingen, Germany).

All experimental measurements were performed using an Agilent GC 7890B-MS 7010 instrument equipped with a PTV-LVI (Programmable Temperature Vaporizer-Large Volume Injector) injection port and an automatic liquid sampler Agilent 7693A. The chromatographic column was an HP-5MS UI 95% dimethyl-5% phenylpolysiloxane (30 m × 0.25 mm, 0.25 μm film thickness). The oven temperature program was as follows: initial temperature 50 °C, isothermal for 5 min; 10 °C min^−1^ up to 150 °C, isothermal for 8 min; 2 °C min^−1^ up to 230 °C, isothermal for 6 min; 10 °C min^−1^ up to 300 °C and isothermal for 24 min.

The injector was at an initial temperature of 55 °C with the splitting valve open for 30 s for the evaporation of the solvent. Then it was closed, and the temperature increased up to 280 °C with a speed of 500 °C min^−1^. The carrier gas was ultrapure helium, with an initial pressure of 79 Pa.

Sediment samples were thawed overnight at room temperature, always closed in their aluminum packaging in order to avoid any possible contamination, in a clean room ISO 5. About 2 or 3 g of sediment were placed in a ceramic crucible in an oven to dry overnight, at about 110 °C, to have an estimate of the sediment dry weight.

About 25–35 g of the sediment were transferred to a 250 mL glass flask, and both 10 μL of recovery internal standard and 20 mL of a 1:3 hexane:acetone mixture were added; then the flask was placed in a pre-heated ultrasonic bath at 60 °C for 30 min. After this treatment, the supernatant liquid was withdrawn and placed in a 25 mL glass vial. The ultrasonic bath treatment was repeated a second time after adding a second 20 mL aliquot of the hexane-acetone mixture.

The extracts were combined and placed in a centrifugal evaporator to reach a volume of about 1 mL of extract. Four milliliters of isooctane were added and re-evaporated until 2 mL of extract was obtained.

The extracted sample was purified from elemental sulfur with tetrabutylammonium sulfite. TBA solution was prepared by adding 1.696 g of TBA and about 12.5 g of sodium sulfite to 50 mL of double-distilled water until saturation [19,20].

For this procedure, 1 mL of propanol, 1 mL of TBA solution, and 5 mL of double distilled water were added to the extracted sample; the solution was stirred for about 1 min and then left to rest for 10 min to separate organic and aqueous phases [21]. The organic phase was purified on a Florisil cartridge (Supelco) to separate non- or less-polar analytes from polar interfering compounds before chromatographic analysis.

At the end of the purification stage, samples were concentrated by means of the centrifugal evaporator up to a volume of about 1 mL, controlled by weighing, and 10 μL of injection standard was added to each sample before injection into the gas chromatograph.

### 2.5. Estimation of Microbial Cell Abundance, Biomass, and Enzymatic Activities

#### 2.5.1. Total Cell Count by Flow Cytometry

The abundance of prokaryotic cells was estimated by following cell detachment from sediment and counting procedures, as described elsewhere [22,23]. Briefly, wet sediment (1 g) was suspended in PBS (1×), amended with sodium pyrophosphate (1 g L^−1^, final concentration, Sigma-Aldrich) and Tween20 (0.5%, final concentration), shaken vigorously (30 min, 720 rpm; IKA^®^ KS 130 B, Staufen, Germany), and sonicated (1 min, 20 W; Microson XL2000 ultrasonic liquid processor, Misonix, New York, NY, USA). Aliquots of supernatant were fixed with formaldehyde (2% final concentration) and kept refrigerated until analyses. Upon staining with SYBR Green I (1:10,000 final concentration; Molecular Probes, Invitrogen), the total cell count (TCC, expressed as cells g^−1^ of wet sediment) was determined in all sediment suspensions by the flow cytometer A50-micro (Apogee Flow System, Hertfordshire, UK) equipped with a 20 mW Solid State Blue Laser (488 nm). A fixed threshold value was set on the green channel to exclude most of the background noise below the 10 fluorescence units. The light scattering signals (forward and side light scatter) were acquired along with green (535/35 nm), orange (590/35 nm), and red fluorescence (>610 nm). TCC was carried out in density plots of the side scatter vs. the green channel. The percentages of cells with low and high nucleic acid content of the TCC were detected at values within 10–100 green fluorescence (LNA cells) and >100 green fluorescence (HNA cells) over the fixed threshold [24]. Samples were run at a low flow rate (1.4 μL min^−1^) in order to keep the number of total events below 1000 events/s. An exclusion gate was applied to avoid the visualization of abiotic particles characterized by low green and high red fluorescence. Apogee Histogram software (version 89.0) was used for data handling and visualization.

#### 2.5.2. Total Prokaryotic Biomass, Cell Volume, and Morphotypes by Image Analysis

Samples for total prokaryotic biomass (PB), cell volume (VOL), and morphotype estimations were fixed with formaldehyde (2% final concentration) and stored in the dark at 4 °C. Samples were diluted with filter-sterilized water to a total volume of 5 mL. The de-tailed methodological procedures were reported in Porter and Feig [25] and La Ferla et al. [26].

#### 2.5.3. Enzymatic Activities

To measure the enzymatic activities (leucine aminopeptidase, LAP; beta-glucosidase, beta-GLU; alkaline phosphatase, AP) expressed by the microbial community, the fluorogenic compound method was applied [27,28]. The sediment samples were diluted in a 1:10 ratio (*w*/*v*) in sterile water and homogenized. The obtained supernatant (0.5 mL sub-volumes) was incubated with volumes (50 to 300 micromoles) of the substrates leucine aminomethylcoumarine (MCA) (Leu-MCA), 4-methylumbelliferyl (MUF)-beta-d-glucopyranoside (MUF-glu), and MUF phosphate (purchased from Merck Life Science S.r.l., Milan, Italy) for LAP, beta-GLU, and AP measurements, respectively. At time 0 (i.e., just after addition of the substrate) and after 1.5 h of incubation at the temperature recorded “in situ”, fluorometric readings were carried out the excitation/emission wavelengths of 380/440 nm for LAP, while for beta-GLU and AP the measurements were performed at 365/445 nm. MCA and MUF were used at known concentrations (200 to 800 nmoles) to build calibration curves for LAP and beta-GLU and AP data. Substrate hydrolysis was reported as the maximum velocity (Vmax) in millimoles of carbon and phosphorus released per gram of sediment and per hour (mmol g^−1^ h^−1^).

### 2.6. Phylogenetic Composition of the Benthic Bacterial Community

#### DNA Extraction and PCR Amplification

DNA extraction was performed from −20 °C-stored sediment sub-samples (approx. 0.5–0.8 g) by the PowerSoil kit (MoBio Laboratories Inc., Biogenerica, Catane, Italy) according to the supplied protocol. The 16S rRNA genes (V1-V2 region) were sequenced using Ion Xpress technology and performed with a previous PCR amplification using the universal primers 27f: 5′-AGAGTTTGATCCTGGCTCAG-3′; 338r: 5′-GCT GCC TCC CGT AGG AGT-3′. Briefly, the extracted DNA was checked and quantified using the Agencourt AMPure XP kit (Beckman Coulter, Inc., Milan, Italy) and the Qubit dsDNA HS Assay Kit (Qubit Fluorometer 2.0, Invitrogen, Thermo Fisher Scientific, Milan, Italy), respectively. The obtained pool of DNA was subsequently sequenced by the Ion Torrent Personal Genome Machine™ using the Ion PGM Sequencing 400 Kit and the Ion 314™ chip (all Ion Torrent reagents by Thermo Fischer Scientific, Milan, Italy) [29].

The resulting reads were checked for quality using Trimmomatic software, and a sliding window 4:20 was chosen to discard low-quality reads [30]. Bioinformatics analysis was performed with Qiime2 version 2020.8. The denoising step was carried out with DADA2, supporting the QIIME2 pipeline [31]. High-quality reads resulted in approximately 42,094 for 15 samples, with an average of 2806 reads (44.9%) per sample. All samples were included in the downstream quantitative analyses. Sequences were taxonomically identified through the SILVA reference files (SILVA release 138 full-length sequences and taxonomy references, release December 2019) using classify-consensusblast. Sequences were clustered into OTUs and taxonomically assigned at 97% identity [31]. The resulting OTU table was normalized to the lowest number of reads among the samples (1865) for subsequent analyses.

### 2.7. Statistical Analyses

The statistical methods included Pearson’s correlation coefficients, principal component analysis (PCA), hierarchical cluster analysis (CA), and non-metric multi-dimensional analysis (nMDS). Statistical analysis was performed on transformed data (log or square root transformation depending on data type) to compare environmental parameters, biological data, and taxonomic abundance values between samples collected in Ice-melt(−) and Ice-melt(+) periods. Results were considered statistically significant when *p* < 0.05. Correlation coefficients of contaminant concentrations vs. environmental parameters and vs. biological data were calculated to establish the occurrence of significant relationships. Euclidean distance was calculated on transformed environmental data obtained at each station during Ice-melt(−) and Ice-melt(+) conditions, then clustering analysis was applied, and a PCA was carried out using the correlation matrix. Similarly, the Bray–Curtis similarity was calculated on transformed biological data, and the similarity matrix was used to compute the nMDS ordination plot. Vectors from inorganic and organic pollutants were superimposed on both PCA and nMDS to observe the grouping of the stations and the correlations with the pollution levels (Primer 7 Plymouth Marine Laboratory, Roborough, UK). Data on heavy metal pollution, as previously obtained for Pasvik River sediment [10], were included in the analyses. Venn diagrams were generated from obtained OTUs (riverine and brackish stations) by using the web-based tool ‘InteractiVenn’ [32].

## 3. Results

### 3.1. Sediment Characteristics

As shown in Table 1, the sand content ranged from 55.5% (at St. 3) to 94.7% (at St. 1) and from 39.8% (at St. 5) to 99.1% (at St. 9) in the Ice-melt(−) and Ice-melt(+) periods, respectively. The mean SAND value was 83.2% in Ice-melt(−) and 73.3% in Ice-melt(+). Sand prevailed at all stations, both in May and July, except at St. 5 (39.8%) and St. 6 in July (48.7%), probably reflecting the river hydrographic regime.

The organic carbon content ranged between 0.12 (at St. 2) and 1.90% (at St. 3) in May and between 0.08% (at St. 9) and 1.34 (at St. 6) in July. The mean C-ORG value was 0.61% in May and 0.71% in July.

A linear relationship (r^2^ = 0.74) was observed between C-ORG and N-TOT contents, considering all surface sediments. It is noteworthy that the x-intercept (%C-ORG = 0) of this regression is 0.0013% N-TOT, which is not significantly different from zero.

C/N molar ratios ranged between 6.8 (at St. 5) and 16.6 (at St. 9) in Ice-melt(−), and between 10.1 (at St. 5) and 20.1 (at St. 7) in Ice-melt(+). The mean C/N molar ratios were 12.5 and 14.2 in May and July, respectively.

The δ^13^C values ranged between −28.60 (at St. 9) and −22.07‰ (at St. 7) in Ice-melt(−), and between −27.07 (at St. 5) and −20.13‰ (at St. 1) in Ice-melt(+). The mean δ^13^C value was −24.4 and −24.59‰ in May and July, respectively.

The lowest δ^13^C values were measured at the innermost stations (−28.60‰ at St. 9 in May and −27.07‰ at St. 5 in July) and the highest ones at the outer stations.

### 3.2. Evaluation of Contamination Level

The ranges of LOD (limit of detection) and LOQ (limit of quantification) were 0.0004–0.0007 and 0.001–00.2, 0.001–0.008 and 0.004–0.02, 0.0002–0.001, and 0.0007–0.004 µg/kg for PAHs, OCPs, and PCBs, respectively. The analytes were grouped according to the expected concentration range [33,34,35], and the corresponding calibration curves were obtained based on the following eight concentration levels:

- ACY, ACE, FLU, PHE, ANT, FLA, PYR, BaA, CRY, BbF, BkF, BaP, IPY, and BPE at 0.02, 0.05, 0.1, 0.2, 0.5, 1.0, 2.0, 5.0 ng mL^−1^;
- All PCBs and OCPs at 0.01, 0.02, 0.05, 0.1, 0.2, 0.5, 1, and 2 ng mL^−1^.

All calibration curves were linear in the observed concentration range, with a value of r^2^ always higher than 0.997.

The overall recovery was calculated for the labeled analytes present in the method-standard solution added to the sample before the extraction. PCBs and OCPs showed quantitative recovery. On the other hand, the recovery of PAHs never exceeded 82%, but with a CV% (5–8%), about half compared to that of the other classes of analytes. Since concentrations should be given on a dry weight basis, the water content of the sediments was determined, and it was 56% on average (*n* = 10). By subtracting the moisture content, the dry weights of the samples were calculated, and these weights were used for the calculations of the analytes.

The results of the contamination level of the Pasvik River sediment are given in Table 2. ∑PAH concentrations were particularly high at St. 1 and 8 (2969.23 and 1267.19 ng/g, respectively) during the Ice-melt(+) period. Among PAHs, benzo[A]pyrene generally increased at riverine stations from the Ice-melt(−) to the Ice-melt(+) period. ∑PCBs was generally higher during the Ice-melt(−) period. The highest ∑PCB marker concentration (i.e., 13.23 ng/g) was recorded at brackish St. 7 during the Ice-melt(−) period.

Similarly, this station was characterized by high levels (not exceeding 4.71 ng/g) of individual PCB congeners (namely PCB101, PCB138, PCB153, and PCB180) congener concentrations (often below the LOD during both periods).

Among pesticides, isodrin generally decreased from the Ice-melt(−) to the Ice-melt(+) period at riverine stations (with the highest value determined at the riverine St. 1 (287.03 ng/g) during the Ice-melt(−) period) and increased at brackish stations (max value 43.58 ng/g at the brackish St. 7). Dieldrin generally decreased at all stations from the Ice-melt(−) to the Ice-melt(+) period. The exception was the riverine St. 5, where it reached 175.30 ng/g during the Ice-melt(+) period. ∑HCH, ∑DDX, and ∑Heptachlor Epoxide were in the range 0.48–39.76, 0.15–16.85, and 0.15–71.71 ng/g, respectively.

### 3.3. Estimation of Microbial Abundances, Biomass, and Enzymatic Activities

#### 3.3.1. Total Cell Counts, Prokaryotic Biomass, and Cell Morphotypes

By reflecting the inherent environmental heterogeneity, the abundance of prokaryotic cells was highly variable, with no significant differences found between sampling sites and periods. Notably, TCC showed a higher variability in brackish than riverine sampling sites (respectively, 3.9–4.3 and 4.1–1.4 × 10^7^ cells g^−1^), and in Ice-melt(+) than in Ice-melt(−) periods (respectively, 4.1–7.0 and 3.9–4.0 × 10^7^ cells g^−1^). The microbial community was largely dominated by LNA cells in all sediment samples (89.2–11.9% of TCC).

Cell length varied between 0.492 and 1.812 µm and width between 0.282 and 0.438 µm. Cell volume ranged from 0.049 to 0.186 µm^3^ with a mean value of 0.087 ± 0.040 µm^3^. In both periods, the mean cell volumes varied in a similar range, with larger cell sizes at the brackish stations (mean value 0.040 ± 0.028 µm^3^) than at the riverine ones (Table 3).

Cell carbon content (CCC), dependent on cell volume, varied between 16 and 50 fg C cell^−1^ with a mean value of 25.5 ± 9.4 fg C cell^−1^ without any significant difference between two environmental conditions. As for cell volume, CCC was higher at the brackish stations than at the riverine ones (about 30 fg C cell^−1^). PB was in the range of 93–3534 µg C g^−1^, with the highest values generally detected at riverine Ice-melt(+) stations.

**Figure 2:**
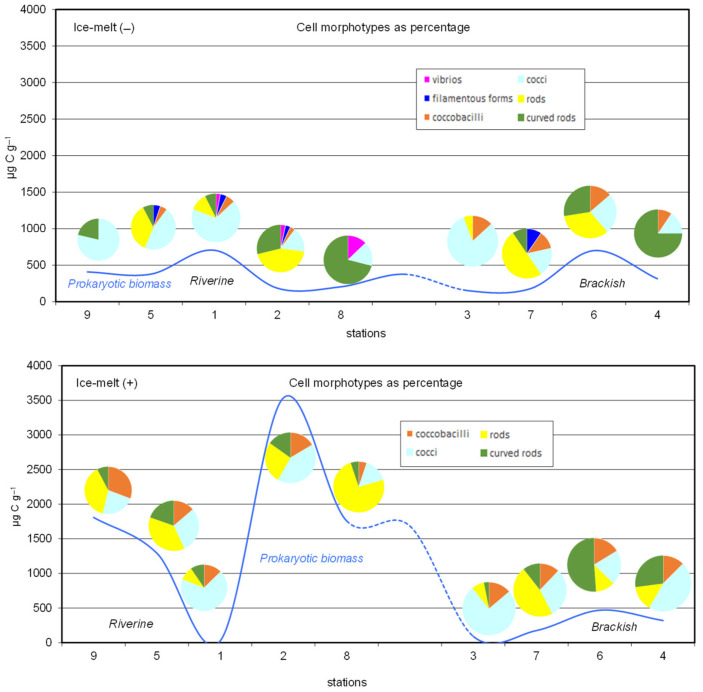
Prokaryotic biomass (blue line) and abundance as a percentage of the total morphotypes at the riverine and brackish stations of the Pasvik River during the Ice-melt(−) and Ice-melt(+) conditions.

Concerning the morphotype composition, the prokaryotic cells were grouped into six classes: vibrios, filamentous forms (i.e., cells exceeding 4 µm in length), coccobacilli, cocci, rods, and curved rods. Rods formed chains and clusters; cocci were found often in colonies and packages. In terms of abundance (Figure 2), cocci dominated the entire dataset, accounting for about 40% of the total cells in both study periods, followed by rods and curved rods. Coccobacilli accounted for 7 and 15% of the total cells in Ice-melt(−) and Ice-melt(+), respectively. Finally, filamentous forms were present only in Ice-melt(−) and at some stations, they contributed to the prokaryotic assemblage to a lesser extent (range: 3–10%).

#### 3.3.2. Microbial Enzymatic Activities

**Figure 3:**
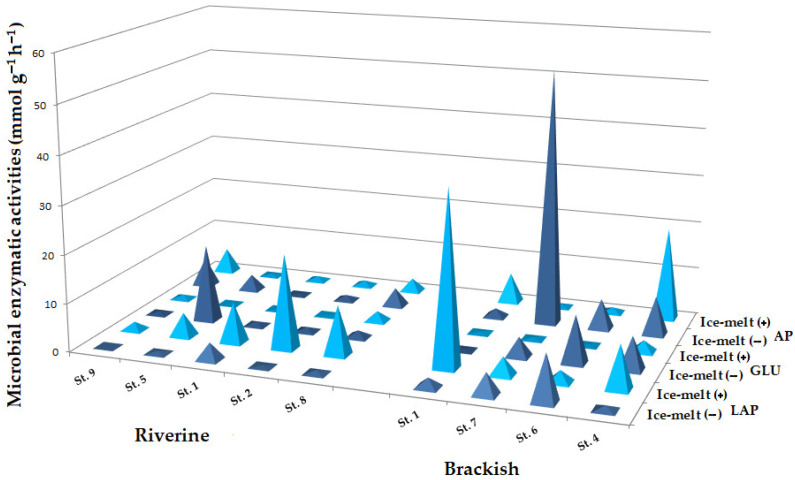
Enzymatic activities in sediment samples from the Pasvik River. Ice-melt(+) and Ice-melt(−) are shown in dark and light blue, respectively.

Microbial enzymatic activities exhibited significant spatial and temporal fluctuations. During the Ice-melt(−) period, LAP rates ranged between 0.02 and 9.73 mmol g^−1^ h^−1^, with the highest values at brackish St. 6 and 7. Enzymatic values generally increased during the Ice-melt(+) period, when they ranged between 1.70 and 36.23 mmol g^−1^ h^−1^. High values of GLU were recorded (0.22–15.98 mmol g^−1^ h^−1^) during the Ice-melt(−) period, particularly at the riverine St. 5 and at the brackish St. 6, 4, and 7; lower values were measured during the Ice-melt(+) period at all stations. Similar to GLU, AP rates decreased in the Ice-melt(+) period (0.47–19.33 mmol g^−1^ h^−1^) compared to the Ice-melt(−) one (0.29–53.39 mmol g^−1^ h^−1^), with peaks at the brackish St. 7 and 4 (Figure 3).

### 3.4. Phylogenetic Composition of the Bacterial Community

**Figure 4:**
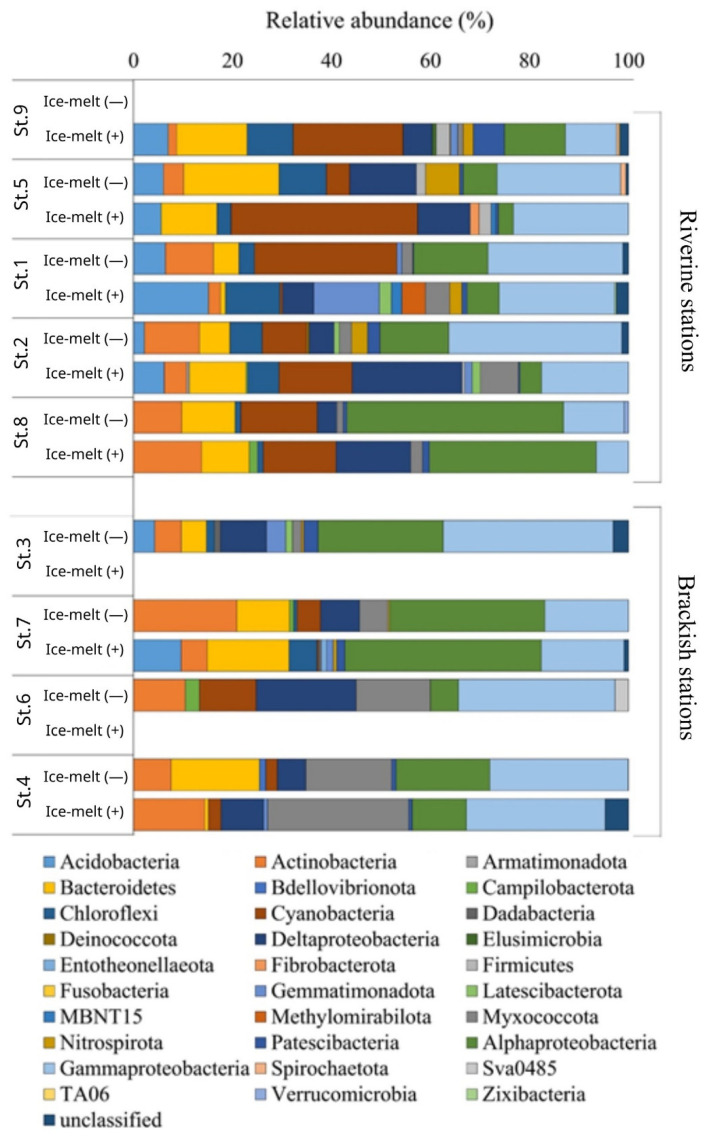
Phylogenetic affiliation of Arctic bacteria retrieved at riverine and brackish stations.

Main data from the bioinformatics analysis (i.e., total sequence reads, quality trimming, OTU information, and diversity indices) are reported in Supplementary Table S1. Overall, Proteobacteria were generally predominant during both ice-melting conditions (range 29.6–78.1% of total community), with higher relative abundances at brackish than riverine stations, as well as during the Ice-melt(−) than Ice-melt(+) conditions. Other major groups were Actinobacteria, Bacteroidetes, and Cyanobacteria (up to 20.8, 19.3, and 28.8% of the total community). Minor groups (i.e., Armatimonadetes, *Deinococcus*/*Thermus*, Elusimicrobia, Fusobacteria, Verrucomicrobia, and Zixibacteria) were sporadically present and occurred at less than 1% of the total community (Figure 4).

In the Ice-melt(−) period, Proteobacteria were mainly represented by Gammaproteobacteria (riverine St. 5, St. 1, and St. 2, and brackish St. 3; range 24.9–34.9% of total community), Alphaproteobacteria (riverine St. 8 and brackish St. 7; 43.0 and 31.4%, respectively) or almost equally dominant Gammaproteobacteria and Deltaproteobacteria (brackish St. 6 and St. 4; approx. 35 and 25% each, respectively; including a moderate portion of Myxococcales; up to 17.4% at the brackish St. 4). Bacteroidetes and Actinobacteria (range 5.1–19.3% and 4.1–20.8%, respectively) accounted for >10% of the total community at several stations (both riverine and brackish). Acidobacteria were better represented (range 2.2–6.4%) at all riverine stations (except for St. 8 where they were absent) and the brackish St. 3 (4.3%). Cyanobacteria (range 2.3–28.8%) followed Proteobacteria in abundance at the riverine St. 1 and St. 8 (28.8 and 15.5%, respectively) and at the brackish St. 6 (11.5%). Other groups contributed to a lesser extent and in a patching pattern to the bacterial community, with percentages between 1 and 5%. Exceptions were Chloroflexi and Nitrospirae at riverine St. 5 (9.6 and 6.8% of the total community, respectively) and Chloroflexi at St. 2 (6.5%).

In the Ice-melt(+) period, Proteobacteria were mainly represented by Gammaproteobacteria (riverine St. 5 and St. 1; approx. 23% of total community) or Alphaproteobacteria (riverine St. 8 and brackish St. 7, 33.7 and 39.7%, respectively). Deltaproteobacteria were particularly abundant at St. 2 and 8 (riverine) (22.2 and 15.1%, respectively), differently for the brackish stations, as they were found only at St. 4 (8.6%); at St. 4 Myxococcales were 28.5%, and they were almost equally dominant with Gammaproteobacteria (28.0%). Gamma- and Alphaproteobacteria were equally dominant only at riverine St. 9 (10.3 and 12.3%, respectively). Bacteroidetes (range 0.8–16.6%) occurred at all stations (both riverine and brackish). The highest abundance of Actinobacteria was determined at riverine St. 8 and brackish St. 4 (13.7 and 14.4%, respectively). Acidobacteria and Chloroflexi occurred at almost all riverine stations (range 5.6–15.1 and 1.1–11.0%, respectively) and the brackish St. 7 (9.6 and 5.6%, respectively). As for the ice-melt(−) period, Cyanobacteria were well represented in sediment samples, following Proteobacteria in abundance at the riverine St. 9, St. 2, and St. 8 (22.2, 14.8, and 14.7%, respectively). Cyanobacteria and Proteobacteria were equally dominant at the riverine St. 5 (approx. 37% of the total community). Other groups contributed to a lesser extent to the bacterial community, with percentages between 1 and 5%. Exceptions were Gemmatimonadetes at the riverine St. 1 (13.2% of the total community) and Patescibacteria at St. 9 (6.3%).

As reported in Table 4, 82 genera occurred at a relative abundance >1%. The highest relative percentages, in the range 12.6–22.7%, were determined for the genera *Woesia* (Gammaproteobacteria), *Loktanella*, and *Pseudorhodobacter* (both Alphaproteobacteria) at a few stations during the Ice-melt(−) period. All other genera occurred at abundances <10% of total community. None of the genera occurred at all stations, while a few genera were retrieved during both periods, e.g., *Illumatobacter*, *Lentimicrobium*, *Paludibaculum*, *Geobacter*, *Bryobacter*, *Terrimonas, Pseudorhodobacter*, *Hydrogenophaga*, *Desulfosarcina*, and *Robiginitomaculum*. In the Ice-melt(−) period, the genera *Mycobacterium*, *Methylocystis*, *Smithella,* and *Massilia* were well represented (between 5 and 10% of total community) at some stations. The same was true for the genera *Rhodoferax*, *Citrifermentans*, *Ferruginibacter*, *Limibaculum,* and *Paracoccus* during the Ice-melt(+) period. Cyanobacteria were particularly abundant at the riverine stations, with *Cyanobium* as the dominant cyanobacterial genus (2.8–7.4% of total sequences). OTU-sharing between Ice-melt(−) and Ice-melt(+) conditions was 27.9% of the total retrieved OTUs (Supplementary Figure S1).

### 3.5. Statistical Analyses

**Figure 5:**
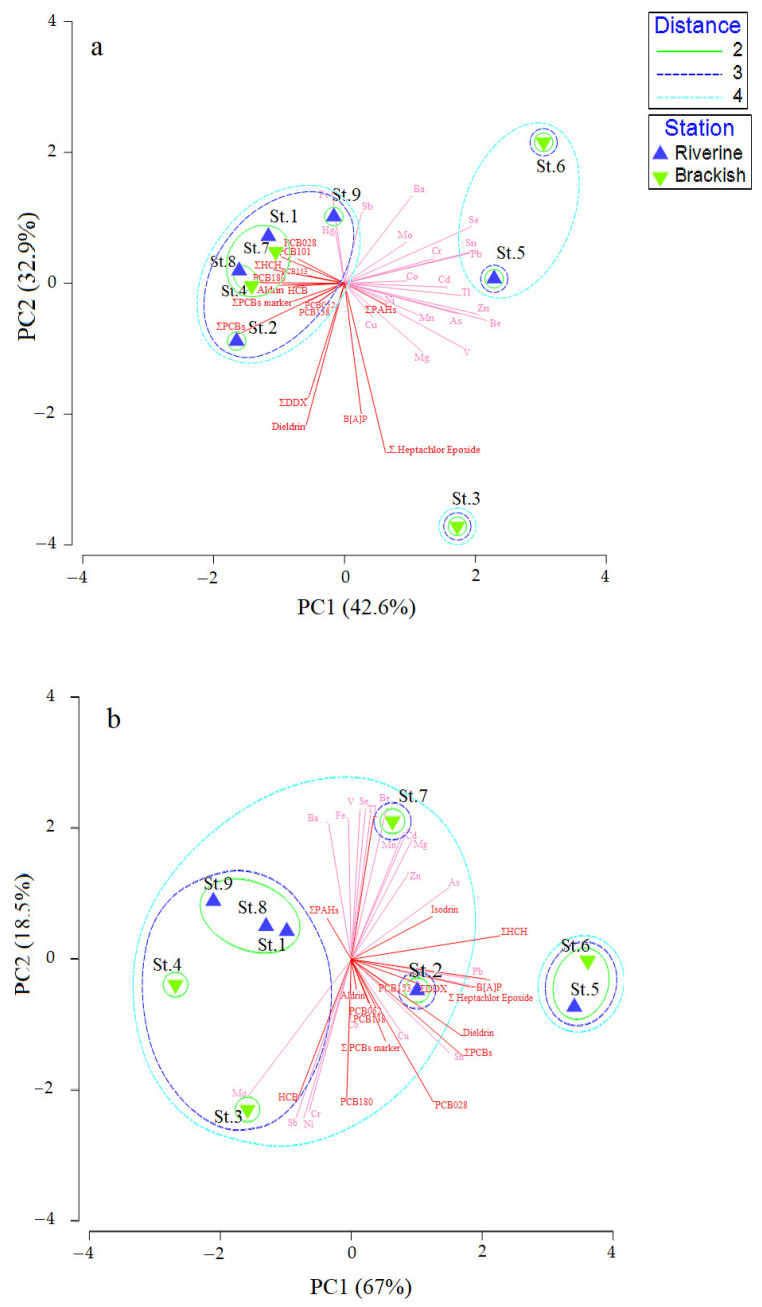
Principal Component Analysis computed on Euclidean distance calculated with environmental data recorded in the Pasvik River during Ice-melt(−) (**a**) and Ice-melt(+) (**b**) conditions. Vectors from inorganic and organic pollutant concentrations have been superimposed (Correlation type Pearson).

The PCA of the physical-chemical parameters of sediments (particulate and texture) and concentrations of contaminants are shown in Figure 5. In the Ice-melt(−) period, the stations St. 2, St. 4, St. 7, St. 8, St. 1, and St. 9 clustered together, with a subcluster including both riverine (St. 8 and St. 1) and brackish (St. 7 and St. 4). A separate group was formed by the riverine St. 5 and the brackish St. 6, while the brackish St. 3 was separated from the remaining stations (Figure 5a). Stations with positive values of the Principal Component PC1 were characterized by high values of metals, while stations with negative values of the PC1 showed high concentrations of organic contaminants. Two main components explained the 42.6% (PC1, mainly influenced by mud and sand, with positive and negative correlation, respectively) and 32.9% (PC2, mainly influenced by CN, Ctot, and Ntot values, with positive correlation) of the total variance (75.5%), respectively. During the Ice-melt(+) period (Figure 5b), St. 5 and St. 6 clustered separately, while all the other stations belonged to a bigger group, in which the riverine St. 9, St. 1, and St. 8 were the most similar to each other.

PC1 and PC2 components explained 85.5% of the total variance: PC1, with a contribution of 67%, was mainly influenced by Ntot and Ctot (positive correlation), while PC2, with a contribution of 18.5%, was mainly influenced by mud (negative correlation) and sand (positive correlation). Here, the stations with positive PC1 values showed high concentrations of some organic pollutants (dieldrin, heptachlor epoxide, DDX, PCB).

The correlation analysis performed on the data referring to the Ice-melt(−) condition highlighted that organic contaminant content (PAHs, PCBs, and pesticides) was not correlated with physicochemical parameters, with the only exception of two correlations found between Heptachlor Epoxide and the Grain Size MUD values (positive correlation, R = 0.850, *p* < 0.05), and the Grain Size SAND values (negative correlation R = −0.850; *p* < 0.05) (Supplementary Table S2). A significant correlation was observed between some heavy metals (i.e., Be, Pb, and Fe) and physicochemical parameters, such as MUD and SAND grain size, Ctot and Cinorg (Be-MUD, R  =  0.745, *p* < 0.05; Fe-C-TOT, R = 0.758, *p* < 0.05; Fe-C-INORG, R = 0.789, *p* < 0.05) (Supplementary Table S3). During Ice-melt(+), according to Pairwise Pearson Correlations, PCBs were positively correlated with MUD (R  >  0.726, *p* < 0.05) and negatively correlated with SAND grain size (R  =  0.726, *p* < 0.05) (Supplementary Table S2), while few significant correlations were evidenced between heavy metals and environmental parameters (Supplementary Table S3).

**Figure 6:**
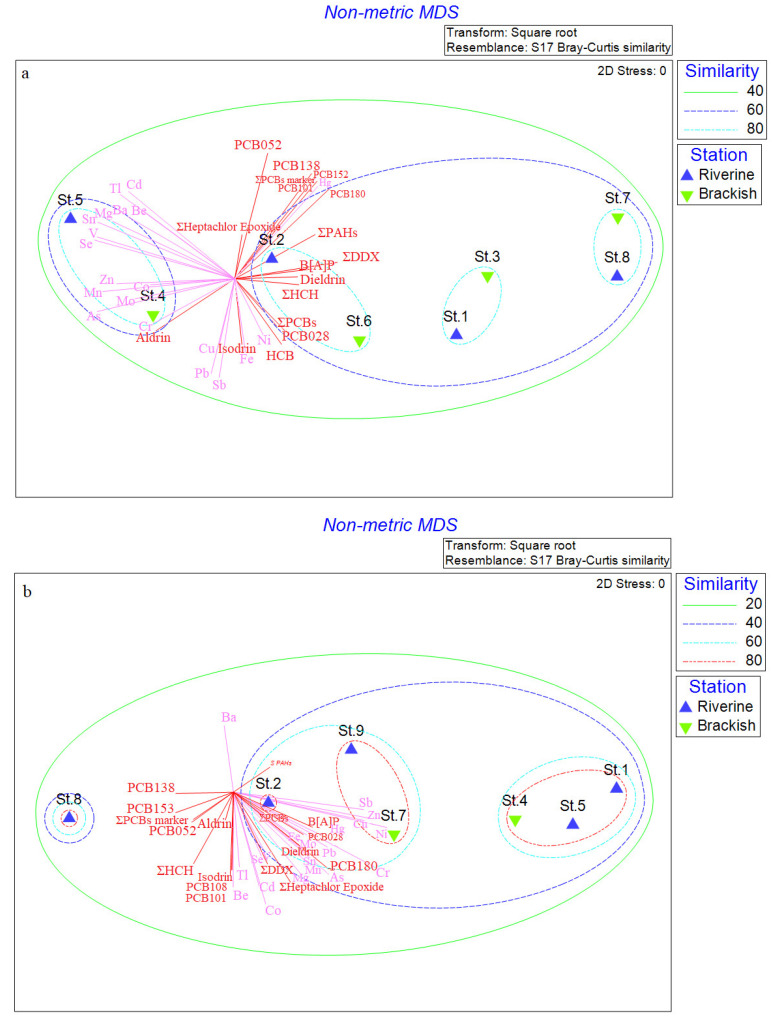
Non-metric multi-dimensional analysis computed microbial parameters recorded from the Pasvik River sediment during Ice-melt(−) (**a**) and Ice-melt(+) (**b**) conditions. Vectors from inorganic and organic pollutant concentrations have been superimposed (Correlation type Pearson).

The nMDS computed on the biological dataset (i.e., total counts, prokaryotic biomass, cell carbon content, morphometry, enzymatic activities, and relative abundance of phylogenetic taxa) is shown in Figure 6. During the Ice-melt(−) season, stations grouped forming two large clusters (60% of similarity): the former included three subclusters with 80% of similarity, composed of St. 2 and St. 6, St. 1 and St. 3, and St. 7 and St. 8; the second cluster included a subcluster formed by St. 4 and St. 5 (80% similarity) (St. 9 was excluded from the analysis) (Figure 6a). Data on the presence of contaminants were superimposed on nMDS, and revealed a strong positive correlation of some stations with organic pollutants (i.e., St. 1, 2, 3, 6, 7, 8), while the others were positively correlated with inorganic pollutants (i.e., St. 4, 5, 9). During the Ice-melt(+) season, two large clusters (40% of similarity) were highlighted. A first cluster included St. 8, and the second one included three subclusters, as follows: St. 1, St. 4, and St. 5 (80% of similarity); St. 7 and St. 9 (80% of similarity); St. 2 (St. 3 and 6 were excluded from analysis) (Figure 6b). As was shown by the overlaid vectors, most of the stations were positively correlated with all metals and some organic pollutants (i.e., heptachlor epoxide, total PCBs, Dieldrin, PCB180, and total DDX).

Pairwise Pearson matrix showed positive correlations (Supplementary Table S4) between the relative abundance of Cyanobacteria and the values of total PCBs and PCB028 (R = 0.799, *p* < 0.05; R = 0.755, *p* = 0.05) during the Ice-melt(−) period, but also between some bacterial taxa and enzymatic activities. This is the case for Deltaproteobacteria, which is positively correlated to LAP (R = 0.780, *p* < 0.05), and Bacteroidetes and Myxococcota, which are positively correlated to beta-GLU (R = 0.884, *p* < 0.05; R = 0.928, *p* < 0.05). Only two PCB congeners, namely PCB052 and PCB180, showed significant correlations with the relative abundance of Firmicutes (R = −0.998, *p* < 0.05) and Myxococcota, respectively (R = 0.947, *p* < 0.05) during the Ice-melt(+) period (Supplementary Table S4).

Significant correlations were evidenced during the Ice-melt(−) between the heavy metal concentrations and the relative abundance of bacterial taxa. Se, Sn, Ba, and Tl were positively correlated with Chloroflexi, while Pb was positively correlated with Deltaproteobacteria. Alphaproteobacteria were positively correlated with Hg concentration (R = 0.731, *p* < 0.05), while Gammaproteobacteria were negatively correlated with the same metal (R = −0.812, *p* < 0.05) (Supplementary Table S5). No significant correlation was evidenced between bacterial taxa and inorganic pollutants during the Ice-melt(+) period, as determined by Pearson’s coefficient values (Supplementary Table S5).

## 4. Discussion

Benthic microbial communities play key roles in the biogeochemical processes of aquatic ecosystems (e.g., energy fluxes, transformation and migration of elements, degradation of pollutants, and self-purification) and in maintaining ecosystem functions [36,37]. Since their structure is highly sensitive to ecosystem stressors, including climate change and contamination, they can be used as bioindicators for monitoring and assessing the disturbance of sediments [37]. However, as highlighted by Wang et al. [38], freshwater riverine sediments have historically been the least studied despite their functional importance, making comparisons among studies difficult. In this study, sediments collected along the Arctic Pasvik River were chemically, biochemically, and microbiologically analyzed.

### 4.1. Chemical Contamination in Sediments of the Pasvik River

Once in aquatic systems, pollutants are partially absorbed by particulate matter, and then localized and accumulated in sediments after settling. Contaminated sediments are of global concern for the possible release of pollutants in water columns. They are a natural repository of pollutants, exerting detrimental effects on living organisms, and may be regarded as a valuable source of information on contamination levels. In riverine systems, sediments are well-known sensitive indicators of environmental and geochemical contamination [39]. The contamination level of sediments from the Pasvik River was evaluated by the determination of PAHs, PCBs, and OCPs. The grain size mostly influenced the distribution of contaminants independently of the thawing processes. PAHs are ubiquitous contaminants of freshwater sediments that can arise from incomplete combustion of organic matter in flames, engines, and industrial processes (pyrogenic PAHs), and from emissions of oil-derived products (petrogenic PAHs). In this study, the levels of ΣPAH in the surface sediments were generally less than 1000 ng g^−1^ dw. The highest concentrations of PAHs were found during the Ice-melt(+) period at both St. 1, next to Nikel, and St. 8 (i.e., more external riverine station), confirming the high dependence of anthropogenic input into PAH levels in sediments. A classification system, based on five different categories derived from ΣPAH concentrations and developed for marine sediments [40], is sometimes applied to freshwater sediments [41,42]. Using this system, the surface sediments from all the examined stations could be classified as “insignificantly contaminated (<300 ng g^−1^ dw)” or “moderately contaminated (300–2000 ng g^−1^ dw)” by PAHs. However, sediments from station 1 are classified as “markedly contaminated (2000–6000 ng g^−1^ dw)”. These results indicate that human sources are predominant. Looking at the concentrations of individual PAHs, it can be concluded that pyrogenic origin might be the dominant PAH source in the Pasvik district. At St. 1, 8, 6, and 4, ice thawing probably caused an increase in PAH concentrations due to the large accumulation of these pollutants during the winter period on the ice sheet, and their transfer into the waters and sediments over the summer months. St. 5 and 2 showed similar PAH concentrations in the May and July samplings, which in any case denotes an increase, albeit less marked, if considering the dilution. This increase might also be explained by the ice formation process. During cold months, ice could act as a contaminant accumulator by entrapping atmospheric particulate material, which is then released, along with the pollutants adsorbed on it, to the water body during melting. Although this effect seems to be limited in time and space, it is nevertheless significant because it happens during summertime, when biological species have their highest activity. In this study, St. 7 was the most polluted station in the brackish area. This finding is justifiable due to the commercial and industrial activities occurring in the Kirkenes area. Similarly, St. 8 showed pollutant concentrations higher than all the other riverine stations (except for St. 1), due to its proximity to the town of Kirkenes.

As far as PCBs are concerned, a general pattern of decreasing PCB levels with increasing distance from the Nickel smelter was observed. In both riverine and brackish areas, the highest values of the low chlorinated PCBs were recorded. This behavior may be justified by the fact that they represent the predominant PCB congeners in Aroclor mixtures (i.e., Aroclor 1016 and Aroclor 1242) previously widely used in cold environments due to their chemical-physical properties. The levels of PCBs in the Pasvik watercourse were higher than those found in surface sediments from lakes in northern Norway [41]. As early as 1997, Skotvold et al. [42] investigated 24 different lakes and detected levels from <1.0 ng g^−1^ dw—14 ng g^−1^ dw of Σ7PCB markers in surface sediments. According to this research, the PCB levels measured in the Pasvik watercourse are classified as either “moderately contaminated” (5–25 ng g^−1^ dw) or “markedly contaminated” (25–100 ng g^−1^ dw). The sum of congeners called “markers” (i.e., PCB 28, 52, 101, 138, 153, and 180) includes about half of all non-dioxin-like PCBs (NDL PCBs) occurring in feed and in food. According to the EFSA (European Food Security Authority) the sum of these PCBs constitutes an adequate indicator of the occurrence of NDL PCBs and the consequent human exposure to them.

OCPs have a strong affinity for suspended particulate matter and afterward settle down in sediments due to low water solubility (from 700 mg L^−1^ to 21,300 mg L^−1^) and high n-octanol/water partition coefficients (logKow) values (3.9–6.2). The high levels of pesticides such as Isodrin, Dieldrin, and Heptaclor Epoxide (a metabolite of Heptachlor), commonly used as insecticides and biocides, may derive from their intensive use for agricultural activity of the place, mainly in the past. DDT was the dominant OC-pesticide in the sediment samples. The highest levels were measured in the area of the smelters and downstream (St. 1, 2, and 8). The levels of ΣDDT detected in this study were high compared to levels measured in sediments from the Arctic and Norway [35]. Hexachlorobenzene (HCB) and chlordanes showed the highest levels close to the smelters. However, the levels of these components were significantly lower than the DDT concentrations. In particular, HCH and Dieldrin were detected in samples from almost all the stations, while Aldrin was generally below the LOD. The distributions of OCPs showed different patterns according to their physicochemical properties: HCHs and HCB showed a quite uniform distribution, pointing out their spread through long-range atmospheric transport. In the areas facing the Kirkenes city, lower but still relevant OCP values were found in the seaport areas. Except for St. 6 and 7, all sites showed higher OCPs concentrations in the Ice-melt(−) period, before the thawing process.

### 4.2. Estimation of Microbial Biomass and Enzymatic Activities

Distribution data on prokaryotic cell abundance, size, biomass, and enzymatic activities are reported for the first time in Pasvik River sediments. A spatial diversification in prokaryotic biomass (with a peak observed at the riverine St. 2) was observed between the riverine and brackish stations during the Ice melt(+) period, whereas biomass values remained quite homogeneous during the Ice melt(−) period. This finding may indicate the potential influence of ice-melting processes on the mixing of microbial communities. Larger cell sizes at brackish than at riverine stations were probably dependent on their different trophic features. Morphometric and morphological alterations in microorganisms may represent sensitive indicators in response to environmental conditions in aquatic ecosystems.

Enzymatic activity profiles highlighted the predominance of AP during the Ice-melt(−) period at brackish stations. This enzyme could be of phytoplanktonic origin. During the Ice-melt(+) period the increase in temperature was associated with a change in the expression of enzyme activities. The prevalence of LAP suggests a higher availability of proteinaceous material, especially at brackish stations, probably related to the accumulation/mobilization of organic matter within the benthic domain. The microbial community inhabiting the Pasvik River appeared to be characterized by metabolic plasticity with functional patterns adapted to fluctuating conditions modulated by seasonal trends.

### 4.3. Main Features of the Bacterial Community along the Pasvik River

Phylogenetically, the benthic bacterial communities in the Pasvik River resembled those reported for sediments and soils of other polar environments [43], with main retrieved groups playing major roles in essential ecosystem functions, from primary production to heterotrophy, and recycling of minerals. Consistent with other studies on freshwater ecosystems [44,45,46], in our samples the core sediment bacterial community (OTUs occurring in at least 50% of analyzed sediment samples) included members of the phyla Proteobacteria, Acidobacteria, Actinobacteria, Chloroflexi, Bacteroidetes, and Cyanobacteria. These latter are commonly reported as abundant members of freshwater ecosystems. Most represented cyanobacterial genera (i.e., *Aphanizomenon*, *Tolypothrix,* and *Cyanobium*) were retrieved only at riverine stations. In particular, *Cyanobium* was the most abundant genus. Together with *Synechococcus* and *Prochlorococcus*, it is among the tiniest primary producers (i.e., picocyanobacteria) playing a pivotal role in fueling many oligotrophic aquatic ecosystems, as the carbon fixed is channeled to higher trophic levels through grazing. *Cyanobium* members proliferate successfully from the poles to the tropics, thanks to their capability to adapt to various environmental conditions [47]. Being coccoid in morphology, they probably strongly contributed to the predominance of cocci in our samples. During Ice-melt(−), Cyanobacteria members were more abundant in the riverine St. 1, where ƩPCBs and Isodrin concentrations were higher than in the other stations. The predominance of Cyanobacteria members was observed in the riverine St. 9 and St. 5 in the Ice-melt(+) period, during which St. 5 also showed the highest concentrations of ƩPCBs and Dieldrin. Among heterotrophs, Proteobacteria (the predominant group in almost all analyzed sediments) is a highly metabolically versatile phylum. The presence of complex organic compounds in river sediments can favor the development of other primary consumers and saprophytic microorganisms, encompassing representatives of the phyla Bacteroidetes and Actinobacteria. The Actinobacteria phylum includes members capable of recycling substances, degrading complex polymers, and removing organic and inorganic pollutants. Bacteroidetes species have a major role in organic matter biodegradation, with the preference of complex, recalcitrant high-molecular weight molecules [48,49]. Acidobacteria members are associated with PCB and petroleum compound degradation.

A relatively high proportion of OTUs were significantly different (the OTU-sharing was below 30%) between the two ice-melt conditions and among stations in terms of detected genera. The most abundant sequences (>10%), which were retrieved during the Ice-melt(−) period at a few stations, were related to biofilm-forming genera (i.e., *Woesia*, *Pseudorhodobacter,* and *Loktanella*), suggesting that slower river waters favored adhesion to sediment particles in May. The biofilm-forming *Woesia* [50], an opportunistic r-strategist, occurred at only two brackish stations. The occurrence of bacteria within the *Pseudorhodobacter* genus is generally reported in marine environments [51]. Recently, Chao et al. [52] found *Pseudorhodobacter* members in batch biofilm reactors, while *Loktanella* is considered a primary bacterial surface colonizer [53]. Moreover, among the less represented genera, *Dechloromonas*, *Nitrospira*, and *Ferruginibacter* are involved in the quorum sensing phenomenon and biofilm formation [54]. *Nitrospira* has been proposed as the initial colonizer in biofilms, providing relatively stable attachment sites to other microorganisms [55]. Other bacteria involved in surface colonization were detected, such as *Granulosicoccus*, *Wynogradskyella*, *Robiginitomaculum,* and *Maribacter* [56,57]. Altogether, these findings strengthened previous observations on the isolation of a high number of biofilm-forming bacteria from samples collected from the Pasvik River [58].

Pasvik River sediments probably experienced anoxic conditions, as strictly anaerobic bacteria were detected, e.g., the denitrifying bacterium *Lentimicrobium*, the putrescine-fermenting *Anaerovorax,* and some acetogenic bacteria (e.g., *Acetobacterium* and *Clostridium*). These latter are a specialized group of strictly anaerobic bacteria that convert carbon dioxide into acetate. Acetogenesis is a significant process in cold anoxic environments with acetogenic bacteria that occur both in anoxic freshwater and marine sediments [59].

The C/N ratio is commonly used to reflect the origin and quality of organic matter in marine sediments [60]. In the study area, the variation ranges of C/N ratio do not differ significantly between the two periods, reflecting the importance of terrestrial organic matter impact on sediments. This result could explain the occurrence of bacterial genera commonly found in soil (e.g., *Bradyrhizobium*, *Bryobacter*, *Solibacter*, *Oryzyhumus*, *Gaiella*, *Rhizobacter*, *Terrimonas*, *Acidibacter*, and *Pseudolabrys*) that could derive from terrestrial input. However, their possible association with submerged roots of aquatic macrophytes, such as *Persicaria amphibia* (L.) (water smartweed; Polygonaceae), which are abundant in the investigated area, cannot be excluded. The stable carbon isotope composition of organic matter (δ^13^C) has been extensively used as an indicator of the relative importance of terrestrial *versus* marine organic matter inputs [61]. The lowest values of δ^13^C that were determined at the innermost stations of the Pasvik River probably reflect a gradient from terrestrial to marine-impacted sediments. This finding was strengthened by the presence of salt-tolerant species or species that are common inhabitants of seawater (e.g., *Limibaculum*, *Wynogradskyella*, *Maribacter*, *Subsaxibacter*, *Marinicella*), which were barely detected at the riverine stations but were enriched at the brackish ones.

### 4.4. Bacteria Involved in the Biodegradation of Organic Pollutants

Both pollutant-degrading and pollutant-insensitive species occurred within the analyzed bacterial communities. Some of the genera detected in the present study were previously associated with the degradation of organic contaminants in diverse environments. For instance, *Ilumatobacter* is among the genera that are enriched in the presence of organic pollutants, such as anilines and phenols, PAHs, and organochlorine pesticides [62]. The occurrence of *Terrimonas* members seemed not to be coupled with benzo[a]pyrene concentration, even if this genus includes benzo[a]pyrene-degrading species [63]. The same consideration could be applied to *Hydrogenophaga* members in the case of PCBs [64]. *Dechloromonas* spp. were previously reported as anaerobic metabolizers of hydrocarbons, including alkanes from C6 to C20. Molina et al. [65] reported on a *Mycobacterium* isolate that was able to mineralize pyrene when it was present as the sole source of carbon and energy. Members of the genus *Mycobacterium* are considered common inhabitants of aquatic environments, including rivers, lakes, ponds, and streams, and they are mostly labeled as “nontuberculous mycobacteria” (NTM) [66]. *Devosia*, *Massilia,* and *Paracoccus*, reported among PAH-degraders [67,68,69], occurred at stations with a moderate/high PAH contamination. Hiraishi et al. [70] reported a *Porphyrobacter* isolate capable of degrading biphenyl and dibenzofuran. In the Pasvik River sediments, such genus was detected at stations that were particularly contaminated by aromatic hydrocarbons. Finally, riverine stations harbored syntrophic deltaproteobacterial degraders in the families Syntrophaceae (i.e., *Smithella* and *Syntrophus*) and Syntrophorhabdaceae (i.e., *Syntrophorhabdus*) implicated in the biodegradation of crude oil (and *n*-alkanes) and the degradation of aromatic compounds, respectively [71,72,73]. Collectively, these data highlight the importance of bacterial diversity in the biotransformation of organic contaminants in the Pasvik River.

### 4.5. Bacteria Involved in Biogeochemical Cycles

The bacterial community includes taxonomic groups playing a major role in the global biogeochemical cycles of elements. Their presence, also in the Ice-melt(−) condition, suggests active carbon, iron, and nitrogen cycling under ice, providing evidence that important metabolic and ecological processes occur seasonally in ice-covered inland freshwater. Bacteria involved in the nitrogen cycle were strongly represented in our samples. As was statistically determined, most of the N was probably associated with organic matter [74]. Denitrification represents a fundamental step in the nitrogen cycle and a major dissimilatory pathway of nitrate removal from river ecosystems [75]. Phylogenetically unrelated denitrifying assemblages can participate in the denitrification process in Pasvik River sediment, as follows. Among Alphaproteobacteria, *Pseudorhodobacter* (facultative anaerobe) is involved in the nitrogen and phosphorus removal processes, while *Bradyrhizobium* spp. grow under oxygen-limiting conditions with nitrate via the denitrification pathway. *Lentimicrobium* (among Bacteroidetes) is a strictly anaerobic, denitrifying bacterium. Among Gammaproteobacteria, *Marinicella* could utilize nitrates produced from the anammox process to enhance higher total nitrogen removal [76], while *Pseudomonas* and *Arenimonas* were also detected denitrifiers. Dissimilatory nitrate reduction to the ammonium (DNRA) pathway probably occurred due to the presence of *Geobacter* and *Ignavibacterium*, as recently reported by Ahamad et al. [77]. Nitrification (the biological conversion of ammonia to nitrite and nitrate) is another fundamental process in the nitrogen cycle. Ammonia and nitrite oxidation are accomplished by several highly specialized nitrifiers. In this study, nitrite-oxidizing bacteria were scattered among Nitrospirae, Proteobacteria, and Chloroflexi. The genus *Nitrospira* includes nitrifiers, oxidizing nitrite, and anaerobic ammonia-oxidizing bacteria that convert ammonia to dinitrogen gas. The occurrence of members of *Nitrospira* and *Nitrosomonas* indicated the presence of ammonia, probably derived from decomposition of animal or plant material. Members of the phylum Chloroflexi are capable of aerobically oxidizing nitrite [78] while reducing nitrate and ferric iron [79].

Methane cycling microorganisms were detected at some Pasvik riverine sites, with potential methanotrophic lineages represented by *Methylocystis* and *Methylomirabilis* (both retrieved exclusively at St. 1) and *Crenothrix* (only found at St. 5). Some *Methylomirabilis* spp. have been reported as capable of nitrite-dependent anaerobic methane oxidation, coupling methane oxidation to the reduction of nitrite to N_2_ [80]. One possible explanation for the high occurrence of methanotrophs might be the transient production of methane in the bottom waters due to the decomposition of the aquatic vegetation (a methane-producing process) that densely occurred in the area (e.g., *Persicaria amphibia* and *Equisetum fluviatile*).

Moreover, several strictly anaerobic or facultative Fe-reducing bacteria (FeRB) were retrieved, raising the possibility that metabolisms related to the iron cycle could play an important role in the river. The ability to use Fe(III) as a terminal electron acceptor is microbiologically widespread in natural systems. For example, *Geobacter* species, together with *Paludibaculum* and *Rhodoferax*, are commonly isolated from a variety of sedimentary environments in which dissimilatory Fe(III) reduction can be significant and coupled with sulfur oxidation (e.g., iron oxide reaction with hydrogen sulfide) [81]. *Anaeromyxobacter* (and *Geobacter*) members are iron-reducing bacteria with nitrogen-fixing activity [82]. *Rhodoferax*, as well as some other members of Comamonadaceae, are known to be involved in the iron cycle [83]. The genus *Ferruginibacter* is often found in freshwater sediments [84] and in relation to heavy metal contamination [85]. Conversely, the occurrence of *Gallionella* members at riverine stations suggested that microaerophilic conditions, probably at the sediment oxic–anoxic interface, might have favored the proliferation of species specialized in iron(II) oxidation (FeOB, Fe-oxidizing bacteria). These findings are consistent with high concentrations of Fe previously determined in Pasvik River sediments [10].

In addition to biogeochemical cycling of elements, anoxygenic photosynthesis (a widely distributed process among Bacteria, such as Chlorobi, Firmicutes, Proteobacteria, Chloroflexi, and Gemmatimonadetes) might occur at Pasvik River riverine stations. Notably, a number of aerobic anoxygenic photoheterotrophic bacteria (AAPB) (e.g., *Erythrobacter*, *Afifella,* and *Porphyrobacter*), containing bacteriochlorophyll a (Bchl a) [86], were detected.

## 5. Conclusions

The composition, diversity, and functional changes of microbial communities in the Pasvik River were influenced by local and diversified anthropogenic sources and discharges. External pollution and environmental variability affected the composition of the bacterial communities, with microorganisms differently involved in the carbon, iron, and nitrogen cycles, as well as in the degradation of organic pollutants and toxic compounds. The biological structure of bacterial communities underwent a shift in the two periods, as evidenced by the different distribution among stations, showing a metabolic plasticity with functional patterns adapted to fluctuating conditions modulated by seasonal trends. However, this result did not seem to be significantly influenced by the chemical contamination pattern; instead, it was mostly dependent on site-specific features. In general, the bacterial diversity in the river benthic domain appeared to be influenced by the microniche conditions of the sediments (i.e., deriving from different physical-chemical conditions or the contamination level), which may favor the communities in these habitats. No influence seemed to be exerted by the location of the sampling sites along the river (i.e., riverine vs. brackish) on the microbial community in terms of main phylogenetic diversity (at the phylum level), morphometry, enzymatic activities, and total counts. This was confirmed by the nMDS analysis showing brackish and riverine stations grouping together into two main subclusters. Conversely, the differences in the abundance of certain taxa (at the genus level) may be due to anthropogenic impact or the contribution of exogenous organic matter at some stations. Unlike previous observations on water microbial communities, the Pasvik benthic communities were likely more stable during seasons, showing a high homogeneity between upland and lowland stations due to the evident melting-induced hydrological connectivity. Further analyses, including a continuous monitoring approach at weekly/monthly basis, are needed to individuate main indicators (both chemical and microbiological) of the Pasvik River status, as well as their relationships and repetitive trends.

## Figures and Tables

**Table 1:** Sediment characteristics in Ice-melt(−) and Ice-melt(+) conditions in the Pasvik River.

Parameter *	Acronym		Riverine Stations					Brackish Stations			
**St. 9**	**St. 5**	**St. 1**	**St. 2**	**St. 8**	**St. 3**	**St. 7**	**St. 6**	**St. 4**
Grain size (%)	MUD **	Ice-melt(−)	13.4	39.0	5.3	5.6	6.4	44.5	6.2	20.4	10.3
		Ice-melt(+)ple	0.9	60.2	16.6	49	12.8	45.4	1.4	51.3	2.5
	SAND **	Ice-melt(−)	86.6	61.0	94.7	94.4	93.6	55.5	93.8	79.6	89.7
		Ice-melt(+)	99.1	39.8	83.4	51	87.2	54.6	98.6	48.7	97.5
Organic C content (%)	C-ORG **	Ice-melt(−)	0.67	0.49	0.54	0.12	0.19	1.90	0.54	0.95	0.12
		Ice-melt(+)	0.08	1.19	0.22	0.64	0.75	nd	0.75	1.34	nd
δ^13^C ‰	D13C	Ice-melt(−)	−28.60	−27.20	−24.35	−24.96	−24.60	−22.65	−22.07	−22.82	−23.08
		Ice-melt(+)	−26.31	−27.07	−20.13	−25.14	−25.09	nd	−24.92	−23.44	nd
Nitrogen content (%)	N-TOT	Ice-melt(−)	0.047	0.085	0.040	nd	0.018	nd	0.044	0.118	0.011
		Ice-melt(+)	nd	0.14	0.02	0.05	nd	nd	0.04	0.1	nd

* nd, not determided; ** Data are from Caputo et al. [10].

**Table 2:** Persistent organic pollutant concentrations (ng g^−1^ dry weight) in sediment samples collected in 2014 during Ice-melt(−) and Ice-melt(+) conditions in the Pasvik River. * <LOD, below the limit of detection.

Persistant Organic Pollutants *			Riverine Stations					Brackish Stations			
			St. 9	St. 5	St. 1	St. 2	St. 8	St. 3	St. 7	St. 6	St. 4
Polycyclic aromatic hydrocarbons (ng g^−1^)	Benzo[A]Pyrene	Ice-melt(−)	3.64	0.55	0.25	0.12	11.19	19.55	0.11	<LOD	0.04
		Ice-melt(+)	0.03	50.76	29.45	<LOD	14.24	0.28	1.21	22.32	0.22
	∑ PAHs	Ice-melt(−)	142.02	192.80	27.74	26.33	681.17	141.90	125.54	38.01	1.80
		Ice-melt(+)	12.99	183.30	2969.23	24.01	1267.19	41.81	87.73	575.02	60.96
Polychlorinated biphenyls (ng g^−1^)	∑ PCB marker	Ice-melt(−)	0.26	1.12	1.43	0.88	0.37	0.99	13.23	<LOD	0.21
		Ice-melt(+)	0.14	0.82	0.41	3.85	0.33	2.02	1.16	0.41	0.41
	∑ PCBs	Ice-melt(−)	12.95	12.13	76.42	6.63	8.48	11.55	20.15	<LOD	5.23
		Ice-melt(+)	5.78	22.94	3.72	19.64	1.32	10.30	4.52	3.56	2.10
	PCB028	Ice-melt(−)	<LOD	0.02	0.65	0.01	<LOD	0.05	0.21	<LOD	0.03
		Ice-melt(+)	0.01	0.15	<LOD	<LOD	<LOD	0.39	<LOD	0.34	<LOD
	PCB052	Ice-melt(−)	<LOD	0.02	0.65	0.01	<LOD	0.10	0.33	<LOD	0.02
		Ice-melt(+)	<LOD	0.08	0.16	0.98	0.05	0.54	0.46	0.07	0.01
	PCB101	Ice-melt(−)	<LOD	<LOD	<LOD	<LOD	<LOD	<LOD	1.64	<LOD	<LOD
		Ice-melt(+)	<LOD	<LOD	<LOD	<LOD	<LOD	<LOD	0.08	<LOD	<LOD
	PCB138	Ice-melt(−)	0.13	0.52	0.22	0.34	0.03	0.51	4.71	<LOD	0.05
		Ice-melt(+)	0.05	<LOD	0.17	2.09	0.15	0.51	0.34	<LOD	0.03
	PCB153	Ice-melt(−)	0.04	0.29	0.12	0.15	0.02	0.17	4.40	<LOD	0.03
		Ice-melt(+)	0.03	0.13	0.08	0.78	0.12	0.16	0.21	<LOD	0.02
	PCB180	Ice-melt(−)	0.07	<LOD	0.24	0.27	0.20	0.16	1.93	<LOD	0.08
		Ice-melt(+)	0.04	0.45	<LOD	<LOD	0.01	0.41	0.08	<LOD	0.34
Pesticides (ng g^−1^)	HCB	Ice-melt(−)	<LOD	<LOD	0.25	0.01	<LOD	0.05	0.04	<LOD	<LOD
		Ice-melt(+)	<LOD	<LOD	<LOD	<LOD	<LOD	0.19	0.02	<LOD	<LOD
	Aldrin	Ice-melt(−)	<LOD	<LOD	<LOD	<LOD	<LOD	<LOD	<LOD	<LOD	0.01
		Ice-melt(+)	0.01	<LOD	<LOD	<LOD	<LOD	0.04	0.03	0.02	<LOD
	Isodrin	Ice-melt(−)	0.28	53.17	287.03	11.85	6.56	2.63	0.16	<LOD	2.56
		Ice-melt(+)	0.15	2.27	0.16	0.98	1.49	24.41	43.58	34.77	0.25
	Dieldrin	Ice-melt(−)	3.24	<LOD	10.18	1.27	2.65	42.80	7.23	<LOD	1.01
		Ice-melt(+)	<LOD	175.30	0.04	<LOD	6.70	31.25	<LOD	<LOD	<LOD
	∑ HCH	Ice-melt(−)	2.17	22.26	39.76	1.83	58.82	1.72	6.46	<LOD	0.86
		Ice-melt(+)	0.48	3.33	2.00	15.47	2.73	7.58	18.54	38.78	0.02
	∑ DDX	Ice-melt(−)	0.15	1.68	2.27	0.76	16.85	8.11	1.84	<LOD	0.28
		Ice-melt(+)	0.71	10.71	8.50	10.84	3.76	7.98	9.70	<LOD	0.28
	∑ Heptachlor Epoxide	Ice-melt(−)	0.15	30.15	5.28	2.93	10.27	71.71	3.52	<LOD	1.72
		Ice-melt(+)	0.17	71.70	0.58	15.56	0.76	21.88	37.63	<LOD	0.18

**Table 3:** Morphometric and morphological traits of prokaryotic cells in sediment samples collected in May (Ice-Melt(*−*)) and July (Ice-Melt(+)) 2014 along the Pasvik River.

Morphometric Traits		Riverine Stations					Brackish Stations			
		St. 9	St. 5	St. 1	St. 2	St. 8	St. 3	St. 7	St. 6	St. 4
Mean length (µm)	Ice-melt(−)	0.615	0.940	0.905	1.294	1.358	0.492	1.573	1.002	1.135
	Ice-melt(+)	0.615	1.065	0.563	0.727	0.929	0.518	0.905	1.812	1.076
Mean width (µm)	Ice-melt(−)	0.387	0.365	0.403	0.338	0.282	0.421	0.438	0.380	0.308
	Ice-melt(+)	0.387	0.369	0.375	0.372	0.331	0.429	0.368	0.393	0.420
Mean volume (µm^3^)	Ice-melt(−)	0.050	0.072	0.076	0.098	0.075	0.059	0.186	0.091	0.068
	Ice-melt(+)	0.072	0.102	0.049	0.059	0.071	0.063	0.079	0.170	0.117
CCC (fg C cell^−1^)	Ice-melt(−)	16	22	22	29	23	18	50	27	21
	Ice-melt(+)	22	29	16	19	22	19	24	46	33
Cocci (%)	Ice-melt(−)	78.7	46.2	67.6	16.9	16.1	81.1	19.0	25.0	15.6
	Ice-melt(+)	23.1	29.4	68.3	41.8	15.4	75.4	30.3	20.9	45.8
Rods (%)	Ice-melt(−)	0.0	35.9	11.8	44.1	0.0	5.7	50.0	33.8	0.0
	Ice-melt(+)	38.5	37.3	9.5	26.6	74.4	7.7	47.0	11.6	14.6
Vibrios (%)	Ice-melt(−)	0.0	0.0	2.9	3.4	12.9	0.0	0.0	0.0	0.0
	Ice-melt(+)	0.0	0.0	0.0	0.0	0.0	0.0	0.0	0.0	0.0
Coccobacilli (%)	Ice-melt(−)	0.0	5.1	5.9	3.4	0.0	13.2	11.9	13.7	9.4
	Ice-melt(+)	30.8	13.7	12.7	16.5	5.1	13.8	12.1	16.3	12.5
Curved rods (%)	Ice-melt(−)	21.3	7.7	7.4	28.8	71.0	0.0	9.5	27.5	75.0
	Ice-melt(+)	7.7	19.6	9.5	15.2	5.1	3.1	10.6	51.2	27.1
Filamentous forms (%)	Ice-melt(−)	0.0	5.1	4.4	3.4	0.0	0.0	9.6	0.0	0.0
	Ice-melt(+)	0.0	0.0	0.0	0.0	0.0	0.0	0.0	0.0	0.0

**Table 4:** Relative abundances of genera (>0.1%) occurring in the Pasvik River.

		Riverine Stations										Brackish Stations							
		St. 9		St. 5		St. 1		St. 2		St. 8		St. 3		St. 7		St. 6		St. 4	
		Ice-Melt Period										Ice-Melt Period							
Phylum	Genus	[−]	[+]	[−]	[+]	[−]	[+]	[−]	[+]	[−]	[+]	[−]	[+]	[−]	[+]	[−]	[+]	[−]	[+]
Acidobacteriota	*Bryobacter*	nd	0.9			2.1	2.9						nd				nd		
	*Solibacter*	nd	1.4										nd				nd		
	*Paludibaculum*	nd	0.8	3.7	2.4				2.6				nd				nd		
	*Blastocatella*	nd											nd		1.9		nd		
	*Ilumatobacter*	nd							2.7	4.8	8.1		nd	8.0	3.0	3.9	nd	4.1	8.4
Actinobacteriota	*Kineococcus*	nd				2.8							nd				nd		
	*Mycobacterium*	nd				5.8		1.6				2.4	nd				nd		
	*Nakamurella*	nd											nd		1.5		nd		
	*Oryzihumus*	nd		4.1			1.7						nd				nd		
	*Gaiella*	nd	0.7					3.0					nd				nd		
Bacteroidetes	*Ferruginibacter*	nd	3.4		4.4			3.4					nd		5.6		nd		
	*Winogradskyella*	nd											nd				nd	8.1	
	*Lentimicrobium*	nd	2.4	4.8	6.8								nd				nd		
	*Lutimonas*	nd								1.3			nd	4.2			nd		
	*Maribacter*	nd											nd	5.4			nd	8.6	
	*Subsaxibacter*	nd											nd		1.5		nd		
	*Terrimonas*	nd	1.1			3.0		2.0		0.2			nd		0.5		nd		
	*Ignavibacterium*	nd		1.4						0.2			nd				nd		
Campylobacterota	*Sulfurovum*	nd							0.4		1.7		nd	0.8		2.8	nd		
Chloroflexi	*Kouleothrix*	nd				1.6							nd				nd		
Cyanobacteria	*Aphanizomenon*	nd	2.8	3.9					1.6	0.2	0.4		nd				nd		
	*Cyanobium*	nd	6.4			7.5			6.0	2.8			nd				nd		
	*Tolypothrix*	nd	0.6			2.9		1.8					nd				nd		
Desulfobacterota	*Desulfatirhabdium*	nd							2.2				nd				nd		
	*Desulfosarcina*	nd								1.8	5.8		nd	1.6			nd	1.7	5.9
	*Desulfobulbus*	nd	1.1		2.6				2.3				nd				nd		
	*Desulfoprunum*	nd			1.3								nd				nd		
	*Citrifermentans*	nd	2.1				6.6						nd				nd		
	*Desulfuromusa*	nd								0.7	1.5	4.5	nd	2.2			nd		
	*Geobacter*	nd		5.3	5.7			5.1	5.3				nd				nd		
	*Smithella*	nd		5.9					3.5				nd				nd		
	*Syntrophus*	nd	1.8						3.3				nd				nd		
	*Syntrophorhabdus*	nd	0.5						1.4				nd				nd		
Firmicutes	*Acetobacterium*	nd		1.9					0.6				nd				nd		
	*Anaerovorax*	nd	1.1										nd				nd		
	*Clostridium*	nd			1.2								nd				nd		
Latescibacterota	*Latescibacterota*	nd					2.5	1.1	1.8			1.4	nd				nd		
Methylomirabilota	*Methylomirabilis*	nd					4.9						nd				nd		
Myxococcota	*Anaeromyxobacter*	nd	1.2			2.2							nd				nd		
	*Phaselicystis*	nd						1.7					nd				nd		
Nitrospirota	*Nitrospira*	nd					2.5	3.2				0.5	nd	0.3	0.7		nd		
Patescibacteria	*Kaiserbacteria*	nd	1.7					1.0		0.7	0.3	1.4	nd		1.7		nd	0.5	0.7
	*Moranbacteria*	nd	1.1	0.7				1.0					nd				nd	0.2	
	*Staskawiczbacteria*	nd	1.6										nd				nd		
Alphaproteobacteria	*Afifella*	nd										2.7	nd				nd		
	*Bradyrhizobium*	nd	4.9										nd				nd		
	*Devosia*	nd											nd		2.6		nd		
	*Erythrobacter*	nd								1.3			nd				nd		
	*Limibaculum*	nd											nd		8.0		nd		
	*Methylocystis*	nd				6.7							nd				nd		
	*Paracoccus*	nd											nd		5.3		nd		
	*Polymorphobacter*	nd	1.5		3.0	2.6							nd				nd		
	*Porphyrobacter*	nd									1.7		nd				nd		
	*Pseudahrensia*	nd											nd	1.5			nd		
	*Pseudolabrys*	nd						3.6					nd				nd		
	*Pseudorhodobacter*	nd	0.2							11.6	7.5		nd				nd		
	*Robiginitomaculum*	nd											nd				nd	1.4	3.0
	*Roseomonas*	nd									1.5		nd				nd		
	*Sphingopyxis*	nd											nd		1.5		nd		
	*Sphingorhabdus*	nd		2.7						1.7			nd	1.2	1.3		nd		
	*Yoonia-Loktanella*	nd											nd	16.5			nd		
Gammaproteobacteria	*Acidibacter*	nd				1.1		1.2					nd				nd		
	*Arenimonas*	nd				1.2					0.6		nd				nd		
	*Crenothrix*	nd		3.7									nd				nd		
	*Dechloromonas*	nd							1.9				nd				nd		
	*Gallionella*	nd			1.7			4.8					nd				nd		
	*Granulosicoccus*	nd											nd	3.3			nd		
	*Halioglobus*	nd								1.8			nd				nd		
	*Hydrogenophaga*	nd								1.7	3.3		nd		2.4		nd		
	*Marinicella*	nd											nd				nd	5.6	
	*Massilia*	nd				5.0							nd				nd		
	*Nitrosomonas*	nd										2.3	nd				nd		1.3
	*Porticoccus*	nd		1.5									nd				nd		
	*Pseudomonas*	nd											nd		2.5		nd		
	*Psychromonas*	nd							1.2				nd				nd		4.2
	*Rhizobacter*	nd	3.5						2.3				nd				nd		
	*Rhodoferax*	nd			6.6								nd				nd		
	*Woeseia*	nd										22.7	nd	12.6			nd		

## Data Availability

Data are available upon request. Ion Torrent sequence data obtained from this study were registered as NCBI Bioproject PRJNA728045.

## Supplementary Materials

The following supporting information can be downloaded at: https://www.mdpi.com/article/10.3390/microorganisms10051022/s1, Supplementary Figure S1: Venn showing OTUs shared between Ice-melt(–) and Ice-melt(+) periods; Supplementary Table S1: Next-generation sequencing results, number of good quality reads, OTUs, and diversity indices obtained from data analysis for sediment samples collected during Ice-melt(−) and Ice-melt(+) periods; Supplementary Table S2: Pairwise Pearson Correlations (for most significant value) among the organic pollutants and physicochemical parameters of sediments during the Ice-melt(−) and Ice-melt(+) period. The sample size is given in brackets, and the *p* value is given in italics; Supplementary Table S3: Pairwise Pearson Correlations (for most significant values) among the inorganic pollutants and physicochemical parameters of sediments during the Ice-melt(−) and Ice-melt(+) periods. The sample size is given in brackets, and the *p* value is given in italics; Supplementary Table S4: Pairwise Pearson Correlations (for most significant values) among the organic pollutants and bio-logical parameters of sediments during the Ice-melt(−) period and Ice-melt(+) period. The sample size is given in brackets, and the *p* value is given in italics; Supplementary Table S5: Pairwise Pearson Correlations (for most significant values) among the inorganic pollutants and biological parameters of sediments during the Ice-melt(−) period and Ice-melt(+) period. The sample size is given in brackets, and the *p* value is given in italics.
