# Supplementary material for: Benthic Microbial Communities in a Seasonally Ice-Covered Sub-Arctic River (Pasvik River, Norway) Are Shaped by Site-Specific Environmental Conditions
Source: Microorganisms. 2022 May 12;10(5):1022. doi: 10.3390/microorganisms10051022 (PMC9147904; doi:10.3390/microorganisms10051022)
Supplement: Supplementary file 1 [file microorganisms-10-01022-s001.zip › microorganisms-1684152-supplementary.pdf]

Supplementary Materials:

Supplementary Figure S1. *Venn showing OTUs shared between Ice-melt [-] and Ice-melt [+] periods.*

Supplementary Table S1. *Next-generation sequencing results, number of good quality reads, OTUs and diversity indices obtained from data analysis for sediment samples collected during Ice-melt(-) and Ice-melt(+) periods. Figure S3: Venn diagrams representing OTU-sharing between samples: a) riverine stations in May; b) riverine stations in July; c) brackish stations in May; d) brackish stations in July.*

Supplementary Table S2. *Pairwise Pearson Correlations (for most significative value) among the organic pollutants and physico-chemical parameters of sediments during the Ice-melt(-) and Ice-melt(+) period. The sample size is given in brackets, and the p value is given in italic.*

Supplementary Table S3. *Pairwise Pearson Correlations (for most significative values) among the inorganic pollutants and physico-chemical parameters of sediments during the Ice-melt(-) and Ice-melt(+) period. The sample size is given in brackets, and the p value is given in italic.*

Supplementary Table S4. *Pairwise Pearson Correlations (for most significative values) among the organic pollutants and bio-logical parameters of sediments during the Ice-melt(-) period and Ice-melt(+) period. The sample size is given in brackets, and the p value is given in italic.*

Supplementary Table S5. *Pairwise Pearson Correlations (for most significative values) among the inorganic pollutants and bio-logical parameters of sediments during the Ice-melt(-) period and Ice-melt(+) period. The sample size is given in brackets, and the p value is given in italic.*

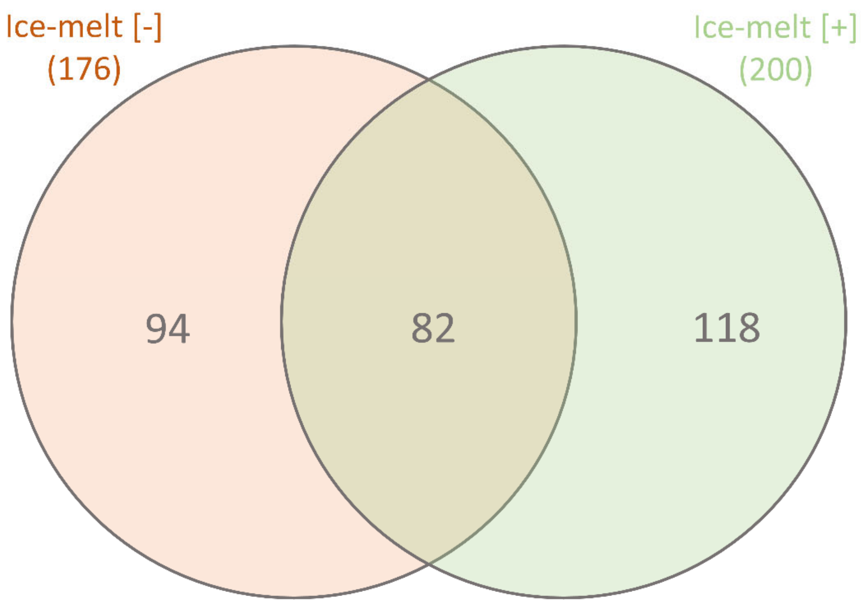

Supplementary Figure S1. Venn showing OTUs shared between Ice-melt [-] and Ice-melt [+] periods.

Supplementary Table S1. Next-generation sequencing results, number of good quality reads, OTUs and diversity indices obtained from data analysis for sediment samples collected during Ice-melt(-) and Ice-melt(+) periods.

		Riverine Stations					*Brackish Stations*			
		St.9	St.5	St.1	St.2	St.8	St.3	St.7	St.6	St.4
Total Reads	Ice-melt(-)	nd	7223	6274	7274	7382	6722	4987	4005	5850
	Ice-melt(+)	6896	6511	5712	9474	6218	nd	4950	nd	4600
GC (%)	Ice-melt(-)	nd	55	55	57	53	55	53	53	53
	Ice-melt(+)	54	54	55	54	53	nd	55	nd	54
Good quality reads (%)	Ice-melt(-)	nd	32.8	45.1	45.2	56.5	41.8	51.4	46.6	42.6
	Ice-melt(+)	46.2	44.6	34.2	43.7	48.2	nd	44.8	nd	50.1
OTUs	Ice-melt(-)	nd	34	31	38	49	34	37	14	25
	Ice-melt(+)	72	26	35	39	38	nd	53	nd	18
Shannon	Ice-melt(-)	nd	2.322	2.22	2.373	2.196	2.196	2.225	1.881	1.968
	Ice-melt(+)	2.603	2.121	2.309	2.376	2.255	nd	2.493	nd	1.903

Inv. Simpson	Ice-melt(-)	nd	0.7387	0.7336	0.7392	0.726	0.7287	0.7328	0.7209	0.7218
	Ice-melt(+)	0.7416	0.7295	0.7372	0.7394	0.7331	nd	0.7408	nd	0.7194
Evenness	Ice-melt(-)	nd	0.2914	0.2877	0.2752	0.1798	0.2569	0.2434	0.4371	0.2754
	Ice-melt(+)	0.1851	0.3089	0.2796	0.269	0.2444	nd	0.2239	nd	0.3528

Supplementary Table S2. Pairwise Pearson Correlations (for most significative value) among the organic pollutants and physico-chemical parameters of sediments during the *Ice-melt(-)* and *Ice-melt(+)* period. The sample size is given in brackets, and the p value is given in italic.

Sample 1	Sample 2	Correlation	95% CI for ϱ	P-Value
		Ice-melt(-)		
C-ORG	Dieldrin	0.853	(0.435; 0.968)	*0.003*
CN	Dieldrin	0.740	(-0.030; 0.959)	*0.057*
ΣDDX	S HCH	0.710	(0.087; 0.934)	*0.032*
MUD	ΣHeptachlor Epoxide	0.850	(0.427; 0.968)	*0.004*
SAND	ΣHeptachlor Epoxide	-0.850	(-0.968; -0.427)	*0.004*
C-ORG	ΣHeptachlor Epoxide	0.775	(0.228; 0.950)	*0.014*
C-ORG	MUD	0.731	(0.129; 0.939)	*0.025*
CN	MUD	-0.815	(-0.972; -0.159)	*0.026*
C-ORG	SAND	-0.731	(-0.939; -0.129)	*0.025*
CN	SAND	0.815	(0.159; 0.972)	*0.026*
N-TOT	C-ORG	0.848	(0.264; 0.977)	*0.016*
C-TOT	C-ORG	0.877	(0.366; 0.982)	*0.009*
C-INORG	C-ORG	0.714	(-0.084; 0.954)	*0.071*
C-TOT	N-TOT	0.962	(0.760; 0.995)	*0.001*
C-INORG	N-TOT	0.924	(0.561; 0.989)	*0.003*
C-INORG	C-TOT	0.962	(0.761; 0.995)	*0.001*
		Ice-melt(+)		
MUD	ΣPCBs	0.726	(0.120; 0.938)	*0.027*
SAND	ΣPCBs	-0.726	(-0.938; -0.120)	*0.027*
HCB	PCB028	0.670	(0.010; 0.923)	*0.048*
C-TOT	PCB028	0.867	(-0.066; 0.991)	*0.057*
C-INORG	PCB028	0.874	(-0.036; 0.992)	*0.053*
CN	PCB101	0.831	(-0.192; 0.989)	*0.081*
CN	Aldrin	0.914	(0.162; 0.994)	*0.030*
ΣHCH	Isodrin	0.738	(0.144; 0.941)	*0.023*
CN	Isodrin	0.871	(-0.050; 0.991)	*0.055*
C-ORG	MUD	0.712	(-0.088; 0.954)	*0.073*
CN	SAND	0.549	(-0.646; 0.964)	*0.338*
C-INORG	SAND	-0.351	(-0.942; 0.770)	*0.563*
D13C	C-ORG	-0.249	(-0.844; 0.620)	*0.591*
N-TOT	C-ORG	0.879	(-0.016; 0.992)	*0.050*
C-TOT	C-ORG	0.997	(0.951; 1.000)	*0.000*
CN	C-ORG	0.089	(-0.861; 0.900)	*0.887*
C-INORG	C-ORG	0.930	(0.263; 0.995)	*0.022*
C-TOT	N-TOT	0.857	(-0.102; 0.990)	*0.063*
C-INORG	C-TOT	0.956	(0.470; 0.997)	*0.011*

Supplementary Table S3. Pairwise Pearson Correlations (for most significative values) among the inorganic pollutants and physico-chemical parameters of sediments during the *Ice-melt(-)* and *Ice-melt(+)* period. The sample size is given in brackets, and the p value is given in italic.

Sample 1	Sample 2	Correlation	95% CI for ρ	p-Value
		Ice-melt (-)		
MUD	Be	0.745	(0.160; 0.943)	*0.021*
Sb	Cr	0.998	(0.991; 1.000)	*0.000*
Ba	Cr	0.992	(0.962; 0.998)	*0.000*
Hg	Cr	0.996	(0.980; 0.999)	*0.000*
D13C	Cr	-0.705	(-0.933; -0.077)	*0.034*
D13C	Co	-0.732	(-0.939; -0.131)	*0.025*
D13C	Ni	-0.676	(-0.925; -0.021)	*0.046*
CN	Se	-0.898	(-0.985; -0.449)	*0.006*
D13C	Mo	-0.707	(-0.933; -0.080)	*0.033*
MUD	Cd	0.586	(-0.129; 0.900)	*0.098*
SAND	Cd	-0.586	(-0.900; 0.129)	*0.098*
CN	Cd	-0.732	(-0.957; 0.048)	*0.062*
CN	Sn	-0.810	(-0.971; -0.145)	*0.027*
D13C	Sb	-0.679	(-0.926; -0.027)	*0.044*
D13C	Ba	-0.777	(-0.951; -0.234)	*0.014*
MUD	Tl	0.630	(-0.058; 0.912)	*0.069*
SAND	Tl	-0.630	(-0.912; 0.058)	*0.069*
CN	Tl	-0.757	(-0.962; -0.008)	*0.049*
N-TOT	Pb	0.728	(-0.054; 0.957)	*0.063*
C-INORG	Mg	0.349	(-0.548; 0.873)	*0.443*
C-TOT	Fe	0.758	(0.012; 0.962)	*0.048*
C-INORG	Fe	0.789	(0.089; 0.967)	*0.035*
		Ice-melt(+)		
D13C	Sb	0.672	(-0.164; 0.946)	*0.098*
N-TOT	Ba	-0.827	(-0.988; 0.204)	*0.084*

Supplementary Table S4. Pairwise Pearson Correlations (for most significative values) among the organic pollutants and biological parameters of sediments during the *Ice-melt(-)* period and *Ice-melt(+)* period. The sample size is given in brackets, and the p value is given in italic.

Sample 1	Sample 2	Correlation	95% CI for ϱ	p-Value
		Ice-melt(-)		
Deltaproteobacteria	LAP	0.780	(0.065; 0.966)	*0.039*
Bacteroidetes	beta-GLU	0.884	(0.392; 0.983)	*0.008*
Myxococcota	beta-GLU	0.928	(0.582; 0.990)	*0.003*
Chloroflexi	TCC	-0.867	(-0.985; -0.185)	*0.026*
Alphaproteobacteria	TCC	0.838	(0.325; 0.970)	*0.009*
Gammaproteobacteria	TCC	-0.735	(-0.948; -0.062)	*0.038*
ΣPCBs marker	TCC	0.691	(0.050; 0.929)	*0.039*
PCB101	TCC	0.692	(0.052; 0.929)	*0.039*
PCB138	TCC	0.668	(0.007; 0.923)	*0.049*
PCB153	TCC	0.679	(0.028; 0.926)	*0.044*
PCB180	TCC	0.738	(0.146; 0.941)	*0.023*
Myxococcota	HNA	0.875	(0.356; 0.981)	*0.010*
Aldrin	HNA	0.870	(0.488; 0.972)	*0.002*
Aldrin	LNA	-0.870	(-0.972; -0.488)	*0.002*
Myxococcota	Bacteroidetes	0.881	(0.244; 0.987)	*0.020*
Nitrospirota	Chloroflexi	0.979	(0.299; 1.000)	*0.021*
Alphaproteobacteria	Chloroflexi	-0.830	(-0.981; -0.058)	*0.041*
ΣPCBs	Cyanobacteria	0.799	(0.115; 0.969)	*0.031*
PCB028	Cyanobacteria	0.755	(0.005; 0.961)	*0.050*
Alphaproteobacteria	Nitrospirota	-0.954	(-0.999; 0.089)	*0.046*
Isodrin	Nitrospirota	0.968	(0.102; 0.999)	*0.032*
Gammaproteobacteria	Alphaproteobacteria	-0.706	(-0.942; -0.002)	*0.051*
B[A]P	unclassified	0.898	(0.077; 0.993)	*0.038*
ΣDDX	unclassified	0.920	(0.200; 0.995)	*0.027*
		Ice-melt(+)		
Gemmatimonadota	Acidobacteria	0.929	(-0.303; 0.999)	*0.071*
Chloroflexi	Actinobacteria	-0.934	(-0.996; -0.297)	*0.020*
Dieldrin	Cyanobacteria	0.792	(0.097; 0.968)	*0.034*
PCB052	Firmicutes	-0.998	(*; *)	*0.039*
PCB180	Myxococcota	0.947	(0.393; 0.997)	*0.015*

Supplementary Table S5. Pairwise Pearson Correlations (for most significative values) among the inorganic pollutants and biological parameters of sediments during the *Ice-melt(-)* period and *Ice-melt(+)* period. The sample size is given in brackets, and the p value is given in italic.

	Sample 1	Sample 2	Correlation	95% CI for ϱ	p-Value
Ice-melt(-)	Deltaproteobacteria	LAP	0.780	(0.065; 0.966)	*0.039*
	Pb	LAP	0.827	(0.360; 0.962)	*0.006*
	Bacteroidetes	beta-GLU	0.884	(0.392; 0.983)	*0.008*
	Myxococcota	beta-GLU	0.928	(0.582; 0.990)	*0.003*
	V	beta-GLU	0.792	(0.269; 0.954)	*0.011*
	As	beta-GLU	0.702	(0.071; 0.932)	*0.035*
	Se	beta-GLU	0.958	(0.807; 0.991)	*0.000*
	Cd	beta-GLU	0.773	(0.223; 0.950)	*0.015*
	Tl	beta-GLU	0.799	(0.288; 0.956)	*0.010*
	Mg	beta-GLU	0.832	(0.376; 0.964)	*0.005*
	Actinobacteria	AP	0.858	(0.389; 0.974)	*0.006*
	Alphaproteobacteria	TCC	0.838	(0.325; 0.970)	*0.009*
	Gammaproteobacteria	TCC	-0.735	(-0.948; -0.062)	*0.038*
	Myxococcota	HNA	0.875	(0.356; 0.981)	*0.010*
	As	HNA	0.682	(0.033; 0.927)	*0.043*
	Chloroflexi	LNA	0.750	(-0.157; 0.971)	*0.086*
	Mn	LNA	-0.684	(-0.927; -0.037)	*0.042*
	Myxococcota	Bacteroidetes	0.881	(0.244; 0.987)	*0.020*
	Sn	Bacteroidetes	0.782	(0.069; 0.966)	*0.038*
	Mg	Bacteroidetes	0.775	(0.052; 0.965)	*0.041*
	Alphaproteobacteria	Chloroflexi	-0.830	(-0.981; -0.058)	*0.041*
	Se	Chloroflexi	0.860	(0.161; 0.984)	*0.028*
	Sn	Chloroflexi	0.821	(0.029; 0.980)	*0.045*
	Ba	Chloroflexi	0.869	(0.196; 0.986)	*0.025*
	Tl	Chloroflexi	0.802	(-0.028; 0.977)	*0.055*
	Pb	Deltaproteobacteria	0.809	(0.143; 0.971)	*0.028*
	Alphaproteobacteria	Nitrospirota	-0.954	(-0.999; 0.089)	*0.046*
	Ba	Nitrospirota	0.975	(0.216; 0.999)	*0.025*
	Hg	Alphaproteobacteria	0.731	(0.054; 0.948)	*0.039*
	Hg	Gammaproteobacteria	-0.812	(-0.965; -0.252)	*0.014*
Ice-melt(+)	Gemmatimonadota	Acidobacteria	0.929	(-0.303; 0.999)	0.071
	Chloroflexi	Actinobacteria	-0.934	(-0.996; -0.297)	0.020
	PA-IA	Deltaproteobacteria	0.841	(0.093; 0.982)	0.036
	PB	Deltaproteobacteria	0.821	(0.029; 0.980)	0.045
	Zn	TCC	-0.730	(-0.957; 0.051)	0.063
